# Dynamical Analysis of the Regulatory Network Controlling Natural Killer Cells Differentiation

**DOI:** 10.3389/fphys.2018.01029

**Published:** 2018-08-02

**Authors:** Adhemar J. Liquitaya-Montiel, Luis Mendoza

**Affiliations:** ^1^Instituto de Investigaciones Biomédicas, Universidad Nacional Autónoma de México, Ciudad de México, Mexico; ^2^Programa de Doctorado en Ciencias Bioquímicas, Universidad Nacional Autónoma de México, Ciudad de México, Mexico

**Keywords:** NK cells, regulatory network, boolean model, discrete dynamical system, differentiation process

## Abstract

Many disease fighting strategies have focused on the generation of NK cells, since they constitute the main immune barrier against cancer and intracellular pathogens such as viruses. Therefore, a predictive model for the development of NK cells would constitute a useful tool to test several hypotheses regarding the production of these cells during both physiological and pathological conditions. Here, we present a boolean network model that reproduces experimental results reported on the literature regarding the progressive stages of the development of NK cells in wild-type and mutant backgrounds. The model allows for the simulation of different conditions, including extracellular micro-environment as well as the simulation of genetic alterations. It also describes how NK cell differentiation depends on a molecular regulatory network that controls the specification of lymphoid lineages, such as T and B cells, which share a common progenitor with NKs. Furthermore, the study shows that the structure of the regulatory network strongly determines the stability of the expression patterns against perturbations.

## 1. Introduction

NK cells are part of the cell mediated immunity and constitute the main defense barrier against tumorigenic and virus-infected cells in mammals (Herberman et al., 1975; Mandelboim et al., 2001). They promote cell death of their targets through the secretion of cytotoxic enzymes perforin and granzyme B, the release of pro-inflammatory cytokines (such as IFN-γ), and the induction of apoptosis through the expression of FAS ligand or membrane receptor TRAIL (Smyth et al., 2002).

**Figure 1:**
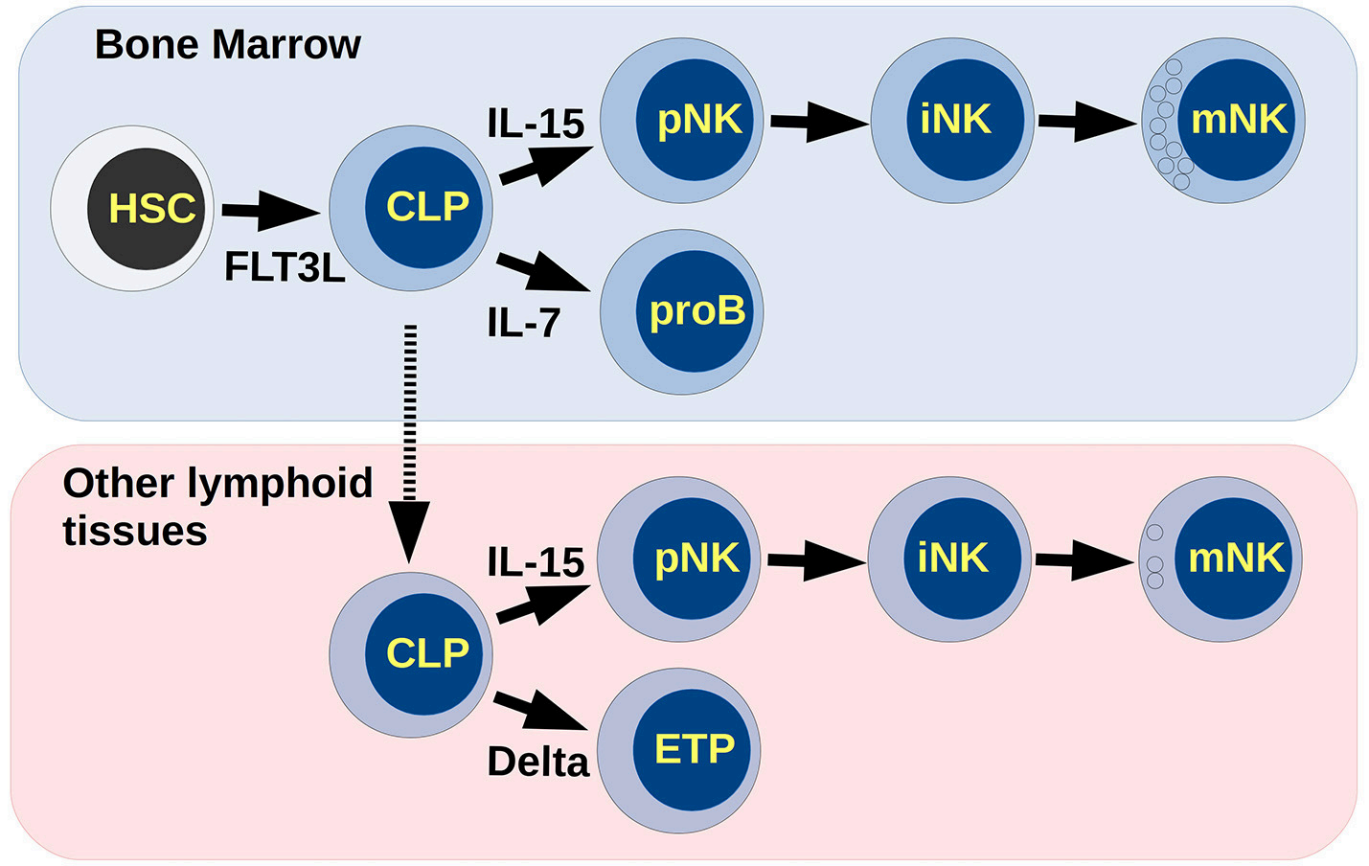
Differentiation of NK cells. Extracellular molecular signals in lymphoid tissues coordinate the differentiation process giving rise to the different lineages of blood cells. Cytotoxic NK cells may appear in the bone marrow, while low cytotoxic and regulatory NK cells may appear in the thymus. Small circles inside mNK cells represent cytotoxic granules.

Hematopoietic stem cells (HSCs) may differentiate to progenitors of the myeloid or the lymphoid lineages. Expression of the transcription factor PU.1 determines the output between these two possibilities. High levels of PU.1 skew the differentiation to the myeloid fate, while low levels determine the appearance of lymphoid-committed cells (Nutt et al., 2005). Common lymphoid progenitors (CLP) give rise to T, B, and NK lymphocytes, depending on the presence of specific molecular signals. Relevant to this process are: (1) Flt3-L, which promotes the progression of lymphoid commitment, (2) Il-7, involved in the differentiation of the B lineage, (3) Notch ligands, which mainly trigger T-cell differentiation, and (4) Il-15, which induces NK cell development *in vivo*, and *in vitro* (Deftos and Bevan, 2000; Sitnicka et al., 2002; Dias et al., 2005; Williams et al., 1998) (see Figure 1).

Three progressive stages in the differentiation of NK cells, identified under experimental conditions, are characterized by the expression of key genetic regulators. The expression of transcription factors E4BP4 and ETS1 establish the commitment of NK progenitors (pNK) (Gascoyne et al., 2009; Ramirez et al., 2012; Male et al., 2014). Later, suppressor Id2 and transcription factors Tox2 and RUNX3 are activated and lead the cells to the immature stage (iNK) (Levanon et al., 2014; Vong et al., 2014). Finally, the presence of the transcription factors T-bet and Eomes mark the transition from iNK to mature NKs (mNK) (Cruz-Guilloty et al., 2009; Vong et al., 2014). Functional NKs may be cytotoxic or regulatory depending on differentiation site as well as the expression of T-bet and Eomes (Daussy et al., 2014), and are characterized by the production of granzyme B, perforin, and IFN-γ (Boos et al., 2007; Gordon et al., 2012; Luetke-Eversloh et al., 2014).

During the differentiation process, pNKs can share immediate precursors with progenitors of B lymphocytes (proB) or with progenitors of T cells (ETP, for early thymoid progenitor). If this process is carried out entirely in the bone marrow, pNKs share precursors with proB and express transcription factor E4BP4, and in turn induces high Eomes expression, thus becoming mature NK cells (Male et al., 2014). Otherwise, lymphoid progenitors in thymus, or other lymphoid tissues, may differentiate to T lymphocytes or NK cells. These NKs are independent of E4BP4 activation, expressing low Eomes and high T-bet and becoming a different subtype of NK (Crotta et al., 2014). It is currently unknown if these populations exhibit cell plasticity and might interconvert into one another.

A large quantity of experimental data in the literature highlights the relevance of specific molecules, and of some regulatory interactions, involved in the development of NK cells. However, the regulatory network that gives rise to the gene expression patterns found during the NK differentiation process remains unknown. The inference and analysis of the dynamical properties of such regulatory network is necessary to understand the molecular mechanism by which lymphocytes progress from a common precursor to a fully differentiated NK cell. This general approach has been fruitful in the study of related hematopoietic processes such as the specification of myeloid and lymphoid lineages (Collombet et al., 2017), the differentiation of granulocytes and monocytes-derived cells (Ramírez and Mendoza, 2018), the differentiation of T and B lymphocytes (Martínez-Sosa and Mendoza, 2013; Mendoza and Méndez, 2015; Méndez and Mendoza, 2016), as well as T-helper cell plasticity (Abou-Jaoudé et al., 2015).

The dynamical analysis of the NK differentiation regulatory network shows that it was necessary to postulate the existence of four regulatory interactions not yet reported in literature. With the incorporation of these interactions, the model is able to recover stationary states that correspond to HSCs, CLPs, proBs, ETPs, and distinct subpopulations of NK cells. Importantly, the model reproduces the progressive pathway leading from stem cells to mature NK cells. All these results fit with experimentally expression patterns reported not only for wild type but also mutant backgrounds. The model reproduces the reprogramming of T to NK lymphocytes (Li P. et al., 2010), and points to the existence of plasticity between subtypes of NK cells mediated by Il-15R signaling. Finally, we show that the dynamical stability of the genetic expression patterns corresponding to NK cells, while compared against their progenitors, is a property of the structure of the regulatory network.

## 2. Materials and methods

### 2.1. Network inference

To determine the structure of the regulatory network, information supporting regulatory interactions among several genes and their products was searched in the published literature. Additionally, we made use of the information provided by the databases BioGRID (https://thebiogrid.org) (Stark et al., 2006), iHOP (http://www.ihop-net.org/) (Hoffmann and Valencia, 2004), and string-db (https://string-db.org) (Jensen et al., 2009). By manually curating the information, we obtained 114 interactions among 36 molecules. The information used to infer the topology of the network is contained in Supplementary Table 1 and summarized in the section 3.1.

### 2.2. Dynamical analysis of the network

A regulatory network is defined by a set of nodes, representing genes and their products, and edges that connect them, representing regulatory interactions (activations or inhibitions). Each node is described by a variable *x*_*i*_ that takes the value of 0 to represent the inactivation or absence of a molecule, or takes a value of 1 to represent the presence and activity of a molecule. The state of *x*_*i*_ is updated at discrete time steps according to a Boolean function *F*_*i*_ such that *x*_*i*_(*t* + 1) = *F*_*i*_(*x*_1_(*t*), *x*_2_(*t*), …, *x*_*n*_(*t*)), where *x*_1_(*t*), *x*_2_(*t*), …, *x*_*n*_(*t*) is the activation state of the set of regulators of the node *x*_*i*_ at time *t*. The state of the network is the set of values of each node at a given time, *X*(*t*) = *x*_1_(*t*), …, *x*_*n*_(*t*). During a simulation, the initial network state evolves to a fixed point or a cyclic state, also known as an attractor. The full set of *F*_*i*_'s, shown in Supplementary Table 2, is expressed in terms of logic operators. To obtain the full set of steady states, we simulated the dynamic behavior of our model using GINsim software (Chaouiya et al., 2012) and compared with expression patterns expected from bibliographic review. Further, we simulated the effect of all possible single mutants of loss and gain of function by fixing the value of each node to 0 or 1, respectively. We then compared the attractors of these model variants with those of the original (wild type) model. In case of loss of function mutants, simulations were performed starting from a specific cell type resembling experimental conditions found in literature. By contrast, gain of function mutants were simulated starting from all possible initial states. The biological interpretation of results were based on genetic expression patterns extracted from published experimental observations.

Additionally, to study the differentiation progress in response to extracellular signals, we made bit-flip perturbations for all combinations of the 4 input nodes for all attractors of the network. Since an input node represents an extracellular signal (Flt3L, Il-7, Notch-ligand Delta, Il-15), this analysis simulated cell type response to distinct microenvironments.

### 2.3. Steady state stability and robustness analyses

To analyze the stability of steady states, bit-flip perturbations for each node were performed for all steady states in the absence of extracellular signals which could stabilize them (Flt3L=Il-7=Delta=Il-15=0). In order to find transitions to diverse attractors, each perturbation was done 10 times under asynchronous updating; this means that nodes were updated only one at a time, and in random order (Garg et al., 2008).

To evaluate the robustness of the NK network due to the boolean functions used to build the model, we analyzed the attractors of 200,000 networks, each of these including one random perturbation of the functions. In this case, bit-flip perturbations correspond to one change in the vectorial representation of the transition function of one node. We compared the percentage of attractors that are conserved with respect to the wild-type network. Furthermore, we quantified the frequency by which the perturbation of a function generated changes in the steady states.

Differentiation progress due to extracellular signals, genetic perturbations, and functions robustness analyzes were performed using functions from *BoolNet* R package (Müssel et al., 2010).

## 3. Results and discussion

### 3.1. Molecular basis of the regulatory network

In adult bone marrow (BM), HSCs express transcription factor Myb, which prevents differentiation and is associated to maintain cells in proliferation (Volpe et al., 2015). Myb is an upregulator of miR-155, which in turn can inhibit the expression of gene PU.1, a negative regulator of Myb, thus forming a negative feedback loop (Basova et al., 2013). The fine tuning of PU.1 is fundamental for determination of hematopoietic lineages (Lieu and Reddy, 2009). Low concentrations of PU.1 results in common lymphoid progenitors development, while high concentration give rise to common myeloid progenitors (CMP) (Dahl et al., 2006). High concentrations of PU.1 activate CEBPα and EGR1, which are two transcription factors relevant to commitment of CMPs. They have a mutual inhibitory regulation with factors involved in CLP commitment; namely, when the expression of PU.1 is high enough to activate CEBPα and EGR1, it inhibits Flt3 receptor and Irf4, Ikaros, and Gfi-1 transcription factors. On the contrary, low concentrations of PU.1 allow the expression of these factors that regulate early lymphoid progression. A regulatory model related to CLP commitment (Spooner et al., 2009) was incorporated into the network presented here. Additionally, the incorporation of regulations involved in other cell types, such as B and T progenitors, was fundamental to generate a model that provided us with information about decisions for NK differentiation process and the extracellular signals that regulate it.

Lymphoid progenitors, in the presence of Flt3 signaling, express the Il-7 receptor, which positively regulates the connected triad of transcription factors E2A, Ebf1, and Pax5 to induce B cell differentiation, (Nutt and Kee, 2007). Alternatively, CLPs may migrate to thymus and differentiate to Early Thymoid Progenitors (ETP) by the program triggered by Notch signaling. Thus, Notch-ligand Delta activates a gene regulatory circuit suggested by Braunstein and Anderson, the connected triad of transcription factors Notch1, HEB, and Bcl11b, which establish T cell commitment (Braunstein and Anderson, 2011). The programs leading to B or T cell lineages inhibit each other, resulting in the specificity of the differentiation process, excluding a distinct cell phenotype at this stage.

Development of NK cells may start from CLPs in BM and, once committed to NK fate, cells may migrate to the periphery and carry out its later stages in liver and other lymphoid tissues. Also, CLPs may migrate to the thymus and differentiate to NK (Huntington et al., 2007). Initial stages of NK differentiation depend on Il-15 cytokine *in vivo* and *in vitro*. Addition of Il-15 to cell culture is necessary for CLPs to commit to NK progenitors (Carson et al., 1994). This interleukin acts via CD122 (β-chain of Il-15 receptor) and triggers a signaling cascade through kinase PDK-1 which, in turn, induce E4BP4 expression (Yang et al., 2015). Transcription factor E4BP4 promotes the transcription of Id2, an important repressor of transcription factors E2A and HEB, suppressing B and T cell differentiation program, and thus allowing for the NK cell specification and progression of its development (Boos et al., 2007; Schotte et al., 2010). However, Schotte et al. proposed that Id2 and E4BP4 may act synergistically and, reports from Ramírez et al. and Zook et al., observed that Id2 transcription is also promoted by transcription factor ETS1 (Schotte et al., 2010; Ramirez et al., 2012; Zook et al., 2016). An alternative pathway, reported by Grund and collaborators, is the induction of ETS1 by MAPK signaling in response of Il-15R activation (Grund et al., 2005). Thus, ETS1 induce Id2 expression in a pathway that does not require E4BP4 activation. This is consistent with Crotta et al. who reported that, in some tissues other than BM, the generation of NK cells is independent from E4BP4 (Crotta et al., 2014). The inclusion of the negative feedback loop between E4BP4 and ETS1, as suggested by Male in Male and Brady (2014), was not necessary to obtain expression patterns of NK cells. Instead, observations from the model set the possibility that Il-15R triggers two independent pathways where both transcription factors may participate independently. Later, ETS1 induce expression of RUNX3, a transcription factor involved in maturation of cytotoxic cells (Zamisch et al., 2009). Thus, based on the relevance of Tox2 in NK maturation observed by Vong et al. we propose an interaction between ETS1 and Tox2, mentioned in detail in the section 3.1.1.

Transition to the mNK stage requires the activation of transcription factors T-bet and Eomes, which regulate expression of functional molecules and determine subpopulations of NK cells in mice and humans. T-bet is induced by Tox2 and ETS1 and it can be self-regulated, resulting in its stable expression (Kanhere et al., 2012; Ramirez et al., 2012; Vong et al., 2014). Similarly, Eomes is upregulated by E4BP4, RUNX3, and its self-regulation (Cruz-Guilloty et al., 2009; Kidder and Palmer, 2010; Kartikasari et al., 2013; Male et al., 2014). Although there are discrepancies about the origin and functions of subpopulations of NK cells, distinct groups agree that one of the classifications of mNKs is given by the expression pattern of both factors. Finally, T-bet, Eomes, and ETS1 are capable of inducing expression of CD-122, establishing a positive feedback loop where signals of extracellular Il-15 not only activate important regulators, but also ensures self-responsiveness (Intlekofer et al., 2005; Ramirez et al., 2012).

#### 3.1.1. Inference of unreported regulatory interactions

Dynamical behavior of the network, based exclusively on the logical functions determined by manual curation of literature, did not result in expression patterns expected for NK cells, or progenitors (Supplementary Figure 1). Specifically, such model lacks a steady state corresponding to NK cells. Therefore, it was necessary to postulate the existence of four regulatory interactions, which might be direct or indirect, in order to reproduce the observed expression patterns. The proposed interactions are based on biological information, specifically on genetic expression patterns observed in different cell types. These regulatory interactions, therefore, constitute predictions of our modeling effort, and the experimental observations that support them are detailed in the next paragraphs.

***Activation of Tox2 by ETS1*.** A lack of regulation from Tox2 to ETS1 has been reported (Vong et al., 2014), but a possible interaction in the opposite direction has not been evaluated. Since Tox2 shows an expression pattern similar to ETS1 (Vong et al., 2014) and the stabilization of mature NK cells result from T-bet self-regulation after its induction by ETS1 and Tox2, we propose that the activation of Tox2 by ETS1 play an important role in the last stage of NK differentiation.

***Inhibition of Flt3 by CD122*.** We propose a negative feedback between Flt3 and CD122 allowing for the transition from CLP (Flt3^+^) to NK progenitors (CD122^+^) (Rosmaraki et al., 2001). The induction of CD122 due to Flt3L activity has been previously reported (Yu et al., 1998). Then, it was observed that Il-2 signaling counteracts the activity of Flt3 in dendritic cells (Lau-Kilby et al., 2011). As it turns out, Il-2R and Il-15 share CD122 (Grabstein et al., 1994). Therefore, it seems likely that Il-15 might signal through CD122 to suppress the expression of Flt3 or signals downstream it, starting the transition from CLP to NK committed cells.

***Activation of CD122 by Notch1*.** Transient Notch signals induce responsiveness to Il-15 in cells with multi-lineage potential –proB Pax5(−/−)–, leading to NK differentiation (Carotta et al., 2006). As mentioned before, Il-15 signals inside the cell are mainly transduced by CD-122 (Grabstein et al., 1994). In addition, incorporation of this interaction allows the model to reproduce T-cell reprogramming to NK after the suppression of Bcl11b as reported by Li P. et al. (2010).

***Inhibition of Myb by CD122*.** Although the role of Myb is poorly understood in NKs development, it is known that the molecule permits maintenance of the proliferation potential in multi-potent cells. It was shown that Myb inhibition is necessary for the maturation of NK cells, achieved through the activity of miR-15/16 (Sullivan et al., 2015). We propose that a molecule downstream CD122 is a likely candidate to repress the Myb factor, thus giving rise to NK cell development progression.

### 3.2. The NK regulatory network

**Figure 2:**
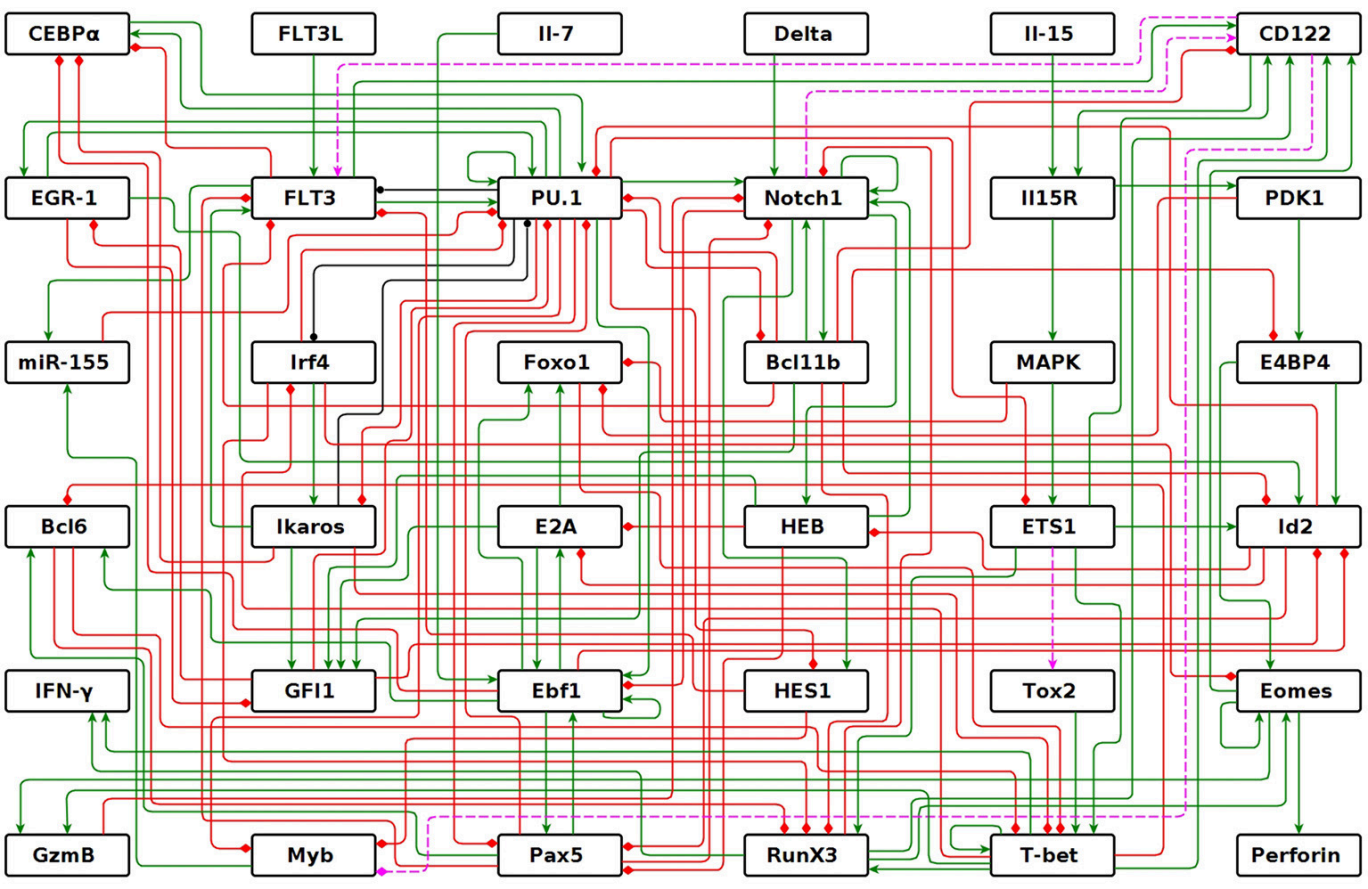
NK regulatory network. Graph of the 36 nodes representing molecules that participate in NK differentiation process and their interactions: activations in green, inhibitions in red, and dual in black. The dotted lines are predictions of the model.

We inferred the genetic regulatory network that modulates differentiation from multi-potent HSCs to mature NK cells. The network consists of 114 interactions—64 activations, 47 inhibitions, and 3 dual (activation or inhibition depending on the context)—among 36 molecules (Figure 2). The network has four nodes that act as inputs: Flt3L, Il-7, Delta, and Il-15. These nodes are used to incorporate, to the model, the signaling of key cytokines present in the microenvironment, allowing for the simulation of extracellular signals found in bone marrow, peripheral lymphoid tissues, or under experimental conditions. The remaining 32 nodes represent different types of molecules that are expressed internally. Specifically: microRNA miR-155; transcription factors Myb, CEBPα, EGR1, PU.1, Irf4, Ikaros, GFI1, Foxo1, E2A, Ebf1, Pax5, Bcl6, Notch1, Bcl11b, HES1, HEB, E4BP4, ETS1, Tox2, RUNX3, T-bet, and Eomes; E-proteins repressor Id2; membrane receptors Flt3, Il-15R, and CD122 (β chain of Il-15R); signaling proteins PDK1 and MAPK; cytotoxic enzymes Perforin and Granzyme B; and cytokine IFN-γ. PU.1 and CD122 have two levels of regulation, therefore, two levels of activation were added in GINsim dynamic model, or two more nodes were added to represent this property for the dynamic analysis of node and function perturbations, with *BoolNet*. The regulatory interactions among these molecules are summarized in Supplementary Table 1.

### 3.3. The regulatory network governs molecular expression patterns during the NK differentiation

Dynamical simulations were performed on the NK network to find all the steady states. The network was implemented as a logical system with the rules for each node as described in Supplementary Table 2. Notice that PU.1 and CD122 were modeled with three values so as to be able to describe three levels of activation; namely, zero, low, and high.

**Figure 3:**
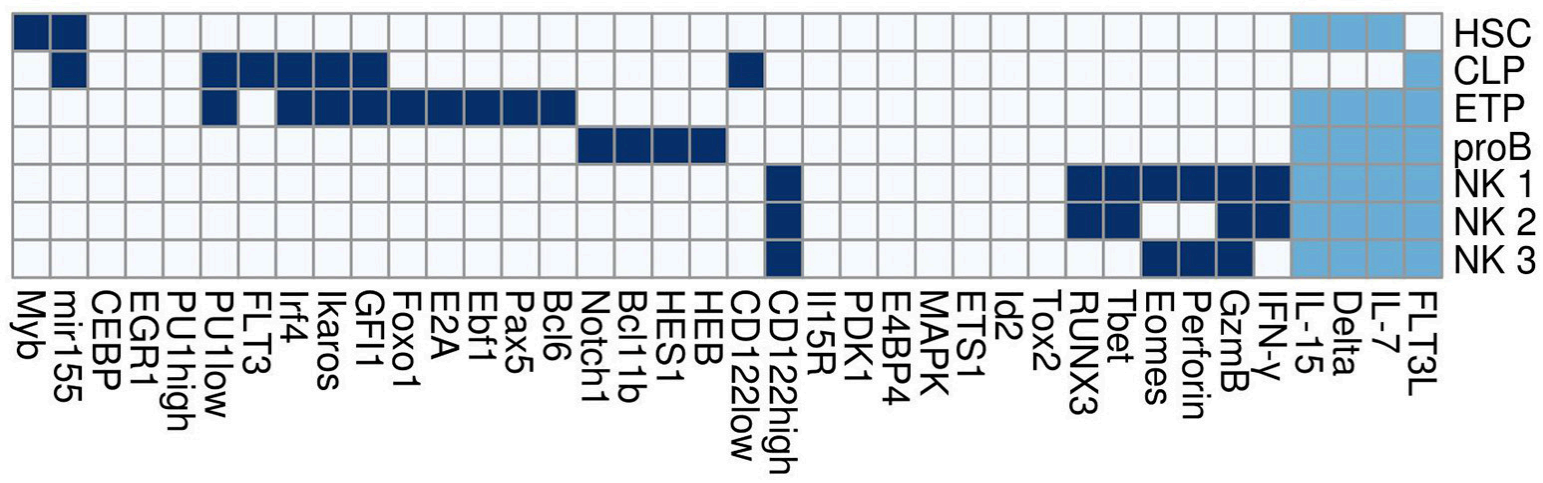
Steady states of the model. Active, inactive, and in any of both states genes are shown as dark blue, white, and light blue squares, respectively. Tags on the columns are the names of the genes. Here, IL-15, Delta, IL-7, and FLT3L are inputs of the network and represent extracellular cytokines. NK 1, NK 2, NK 3, ETP, proB, CLP, and HSC are distinct cell types.

The dynamics of the NK network eventually reaches one of seven cell expression patterns, regardless of the initial state and encompassing the steady states excluding differences due to inputs (Figure 3). Importantly, each of these states correspond to the expression patterns of the cell types during the differentiation of NK cells; namely, HSC, CLP, proB, ETP, and three distinct mature NK cells (T-bet^+^Eomes^+^, T-bet^+^Eomes^−^, and T-bet^−^Eomes^+^). The patterns of the molecular markers used to identify these cell types were extracted from references of Supplementary Table 1, genes that must be present or active are enlisted: Hematopoietic Stem Cell, **HSC:** Myb and miR-155; Common Lymphoid Progenitor, **CLP:** Flt3, PU.1, Irf4, Ikaros and GFI1; B cell committed progenitor, **proB:** PU.1, Irf4, Ikaros, GFI1, E2A, Ebf1, Pax5, Foxo1 and Bcl6; Early Thymoid Progenitor, **ETP:** Notch1, HES1, Bcl11b and HEB; NK T-bet^+^Eomes^+^, **NK1:** CD122, RUNX3, T-bet, Eomes, GzmB, Perforin and IFN-γ; NK T-bet^+^Eomes^−^, **NK2:** CD122, RUNX3, T-bet, GzmB and IFN-γ; and NK T-bet^−^Eomes^+^, **NK3:** Eomes, Perforin and GzmB.

Attractors that correspond to NK cells (NK1, NK2, and NK3) resemble subpopulations observed experimentally in mice and humans(Gordon et al., 2012; Daussy et al., 2014; Knox et al., 2014; Harmon et al., 2016). Although the origin and function of these NK subpopulations are not well established, diverse reports studied the relation among expression of Eomes and T-bet transcription factors to classify NK subtypes. Both factors, in turn, are markers of the different NK related attractors of the network. Peripheral blood NKs in mice and human are T-bet^+^Eomes^−^ and exert high cytotoxicity (Knox et al., 2014). While in human liver there are T-bet^+^Eomes^+^, T-bet^+^Eomes^−^, and T-bet^−^Eomes^+^, this last subtype is enriched and more cytotoxic (Harmon et al., 2016). This is opposite to what is observed in mouse liver, where NKs are mainly T-bet^+^Eomes^−^ being low cytotoxic, while in BM, NKs are T-bet^−^Eomes^+^, with high cytotoxic capacity (Gordon et al., 2012; Daussy et al., 2014).

As mentioned in the introduction, under experimental conditions, pNK and iNK stages are identified as intermediate states of NK differentiation before these cells become mature. Therefore, we analyzed if the model replicates this behavior and searched for pNK and iNK expression patterns as transition states during the simulation. We plotted the average asynchronous transitions of 5,000 simulations using CLP attractor as initial state and simulated addition of Il-15 (Il15 = 1) alongside the simulation (Supplementary Figure 2). After the initial presence of IL-15, the signal passes through IL-15R, thus trigger specific signaling cascades related to NK differentiation. This characteristic constraints the number of possible paths leading to a mature NK. The transition states show a Flt3 decrease, sequential activation and subsequent decrease of E4BP4, Id2, as well as T-bet increase (Supplementary Figure 2A). This sequence corresponds to the transition from stages CLP to pNK, pNK to iNK, and iNK to mNK, respectively. The full set of nodes in the transition of the cell stages is shown in Supplementary Figure 2B.

Of the large number of possible states, namely 2^34^ without considering input nodes, only 7 states are stationary. These stationary states correspond to cell expression patterns observed during the differentiation of NK cells. While these results depend on the set of logical rules used for the specification of the model, these logical rules can be somewhat modified without changing the stationary states (see ahead), strongly suggesting that the stationary states of the network are largely determined by its topology, rather than the specificity of the rules.

### 3.4. Simulation of genetic mutants

Knockout and over-expression mutants were simulated by fixing nodes to 0 or 1, respectively. Results of these simulations are shown in Supplementary Table 3. Note that 16 out of 23 knockouts qualitatively resemble experimental observations, 6 knockout simulations produced no change, and one phenotype has not been experimentally reported in the context of NK cell determination. It is remarkable that the knockout simulation of Pax5, Bcl11b, and HEB reproduce the reprogramming phenomenon to NK cells observed experimentally (Carotta et al., 2006; Li H. et al., 2010; Braunstein and Anderson, 2011). Furthermore, as mentioned in section 3.1, the simulated knockout of either E4BP4 or ETS1 results in the loss of different subtypes of NKs (attractors NK 1 and 3 in case of E4BP4; attractors NK 1 and 2 in case of ETS1), suggesting the existence of two signaling pathways that give rise to distinct subpopulations (see Supplementary Figure 3). This is in accordance with experimental reports where some populations of NK cells, like splenic NK, depend of ETS1 (Barton et al., 1998) and are independent of E4BP4 expression (Crotta et al., 2014), while other NKs depend on E4BP4 (Gascoyne et al., 2009). Thus, two independent pathways triggered by Il-15 result in differentiation to subtypes NK 2 and 3, and are redundant for subtype NK 1. Regarding the simulation of over-expression mutants, 11 simulations reproduce the observed phenotypes, 2 simulations disagree with the reported experimental results, and there are no reports regarding the over-expression of 10 genes in the context of NK cell determination, which allows us to propose the phenotypes of these last as predictions of the model. It is noteworthy that the simulated over-expression of FLT3 resulted in an attractor FLT3^+^RUNX3^+^, which resembles the phenotype of some leukemic cells as shown by Damdinsuren et al. (2015). However, to delve in the study of this disease, more information about regulators related to it must be incorporated.

### 3.5. The model recovers cell progression

**Figure 4:**
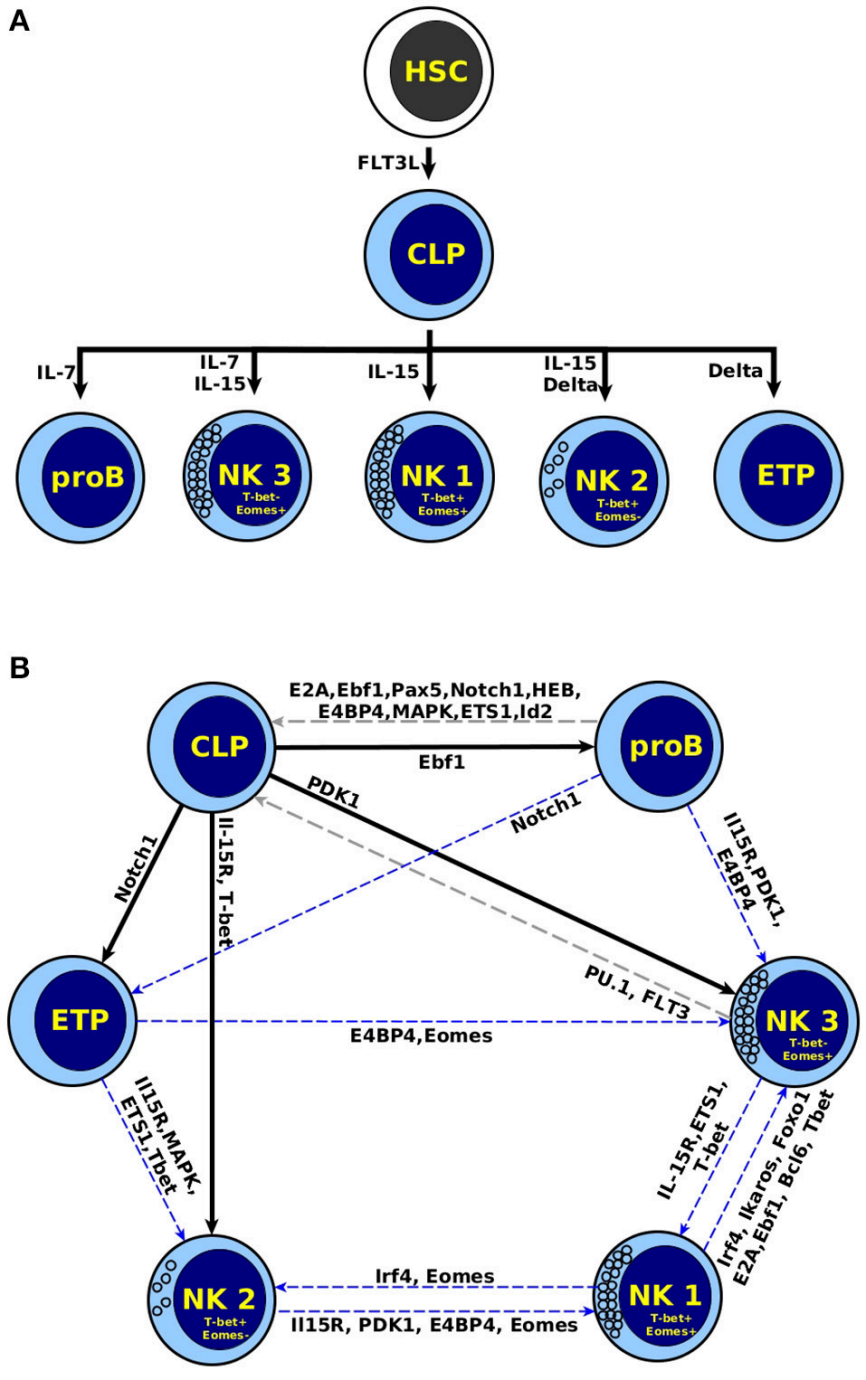
Perturbation of attractors with differentiation signals. **(A)** Inputs representing extracellular signals that regulate NK differentiation (Flt3L, Il-7, Delta, and IL-15) were perturbed in all the steady states. The diagram shows the only five perturbations that result in a change of state, which correspond to biological transitions during the differentiation process. **(B)** Destiny map after perturbing each node. The black arrow corresponds to physiological transitions between cells, the gray dotted line to a transition to a previous state, and the blue dotted line to transition between distinct cell types.

A biological feature of any differentiation process is that the stable expression patterns change in response to specific cues, at the same time cells progressively pass from a state of multi-potency to an increasingly specialized state. This phenomenon does not occur in the reverse direction under physiological conditions. In order to evaluate this biological feature, we simulated the presence of extracellular signals, by means of perturbations of the input nodes when the system is in any of its steady states. As mentioned above, the input nodes in the network represent key cytokines that might be present in the bone marrow (Flt3L, Il-7, and Il-15), and human liver, or mice thymus (Delta and Il-15), which control lymphopoiesis *in vivo* (Beck et al., 2009). The model is able to recapitulate the response of cells to specific cytokines in the microenvironment (Figure 4A). The HSC attractor changes to CLP in response to Flt3L. In turn, CLP changes to proB in response to Il-7, to ETP in response to Delta, and to NK (specifically NK1) in response to Il-15.

The model is also able to describe the appearance of NK subtypes, depending on the combination of input signals. Specifically, the combination of Il-15 and Il-7, which simulates a BM environment, results in the appearance of NK3. These cells are Eomes^+^T-bet^−^, a subtype of NKs enriched in BM of mice and human liver (Gordon et al., 2012; Daussy et al., 2014; Harmon et al., 2016). In turn, Il-15 plus Delta, which resembles the microenvironment in the thymus (Li P. et al., 2010) or liver (Geisler and Strazzabosco, 2015), result in the appearance of NK2. These cells are T-bet^+^Eomes^−^, and can be found in peripheral blood in human (Knox et al., 2014), as well as liver in human and mice (Gordon et al., 2012; Daussy et al., 2014; Harmon et al., 2016).

The generation of NK2 and NK3 in different contexts is in accordance with experimental observations (Daussy et al., 2014), concluding that highly cytotoxic NKs Eomes^+^ are generated in the bone marrow, while regulatory NK T-bet^+^Eomes^−^ found in liver are promoted by Notch signals. Other authors also separate distinct populations of NKs depending on the tissue of differentiation, being bone marrow NK Eomes^*high*^, and pehipheral NKs T-bet^*high*^ Eomes^*low*^ (Simonetta et al., 2016).

### 3.6. Perturbation of genetic expression patterns

The genetic expression patterns used to define a cell type are relatively stable to disturbances. Therefore, we wanted to explore the relative stability of the attractors of the network by performing bit-flip perturbations. This was done by changing the value of one node when the network is in an attractor, and then following its response to evaluate if the temporal perturbation was sufficient to move the network to another attractor or not. This was done in absence of extracellular signals; this is, all input nodes were maintained at 0.

Given that not all possible perturbation are observed under physiological conditions, it is not surprising to observe in the model some transitions that do not occur *in vivo*. The attractors of the model have a varying degree of stability against bit-flip perturbations. Figure 4B shows that only two perturbations change NK3 to CLP. The rest of the perturbations in NK related attractors produce a transition between the three types of NK cells, suggesting the existence of plasticity between these subpopulations. This is relevant because there is a lack of experimental reports on the plasticity between subtypes of NK cells. The existence of transitions among ETP, or proB with NK cells suggest possible reprogramming process due to both attractors correspond to cells at immature stages. Another possibility is that the network lacks some regulatory interactions relevant during T and B cell specification, which are outside the scope of this work. In any case, our model might be useful for pointing to possible experimental treatments inducing a plastic response. Finally, the low number of perturbations that result in transitions shows the relative stability of mature NK cells, as determined by the underlying network.

### 3.7. Dynamical robustness of the network

To asses the robustness of the network, we evaluated to what extent its dynamical behavior depends on the specific selection of the logical rules. We performed random bit-flip perturbations of the Boolean functions and compared the attractors obtained by the perturbed network against those of the original model. The larger the number of attractors shared between the original and the modified versions of the network, the more robust is the original network; in other words, the less dependent is the dynamical behavior of the network on the specific choice of rules. After the simulation of 200,000 perturbations, 64% of them did not produce any change in the steady states compared with the wild type network. The remaining 36% conserved an average of 89.73 ± 0.16% of the total steady states in the network. This indicates that 36% of the changes have repercussion on the attractors, but mostly (89.73%) in the loss of only one cell type. This result is indicative that further characterization of the system likely would show the high redundancy of logical rules on the dynamical behavior of the regulatory network.

The network is to a large degree insensitive to changes in the logical functions that define the response to the regulatory inputs. This indicates that the observed behavior of the network depends on the connectivity of the network, rather than on the use of specific logical rules. This, of course, drastically reduces the possibility of fitting the dynamics of the network by introducing changes into the rules equivalent to parameter fitting. These results strengthen the hypothesis that most of the dynamical properties of the network are due to the connectivity of its nodes.

## 4. Conclusion

We presented a boolean model of the regulatory network that controls the differentiation of NK cells. While there are several models describing different aspects of the differentiation of lymphocytes, to the best of our knowledge this is the first effort to recover the network directly involved in the differentiation of NK cells. The model qualitatively replicates the biological behavior of the process in terms of expression patterns of mature NKs, its progenitor and the related lymphocytes. It also replicates three aspects of biological relevance: (1) the genetic expression of key transitory stages (pNKs and iNKs) from CLP to NK; (2) a high accuracy in replicating the effect of knock out and over-expression mutants; and (3) the progressive transition from a multipotent progenitor to specialized and mature cells.

The model largely lacks parameters to fit, and thus its dynamical behavior is dependent mostly on its connectivity. The incorporation of four specific, and therefore experimentally testable, regulatory interactions resulted in a qualitative description of the model at the molecular level. Such results, also turned out to be very robust, while at the same time they are able to replicate the response to specific extracellular signals coming from the microenvironment. The network model is also able to describe the differentiation of NK subtypes under distinct molecular environments, thus providing a mechanistic explanation for the existence of different subpopulations of NK cells.

It is currently unknown if the cytotoxic and regulatory NK subpopulations are plastic, being able to interconvert into each other. The network model suggests that this is indeed the case, with the possibility of NK cells exhibiting plasticity with the capacity of transdifferentiate under specific conditions, involving IL-15R signals as well as T-bet or Eomes induced expression.

Regarding interaction predictions, the model suggests an outstanding participation of CD122, not only inducing differentiation as it is already known, but also turning off genes important for the differentiation of other cell types. Experimental efforts focused on CD122 downstream factors and its regulation over Flt3, Il-7R, Irf4, Ikaros, and PU.1 might clarify specific aspects of the molecular key events leading a progenitor to become a NK cell.

Finally, the analysis of the dynamical behavior of the network supports the hypothesis that the collective behavior of the molecules included in a regulatory network is highly constrained. The elaboration of a computational model allowed us to study dynamical properties of the regulatory network underlying the differentiation of NK cells, which might pave the way to eventually control it in the laboratory so as to help in the fight against diseases.

## Author contributions

AL-M and LM planned the research, ran the simulations, analyzed the results, and wrote the article.

### Conflict of interest statement

The authors declare that the research was conducted in the absence of any commercial or financial relationships that could be construed as a potential conflict of interest.
