# Supplementary material for: Dynamical Analysis of the Regulatory Network Controlling Natural Killer Cells Differentiation
Source: Front Physiol. 2018 Aug 2;9:1029. doi: 10.3389/fphys.2018.01029 (PMC6082967; doi:10.3389/fphys.2018.01029)
Supplement: Supplementary file 1 [file Data_Sheet_1.PDF]

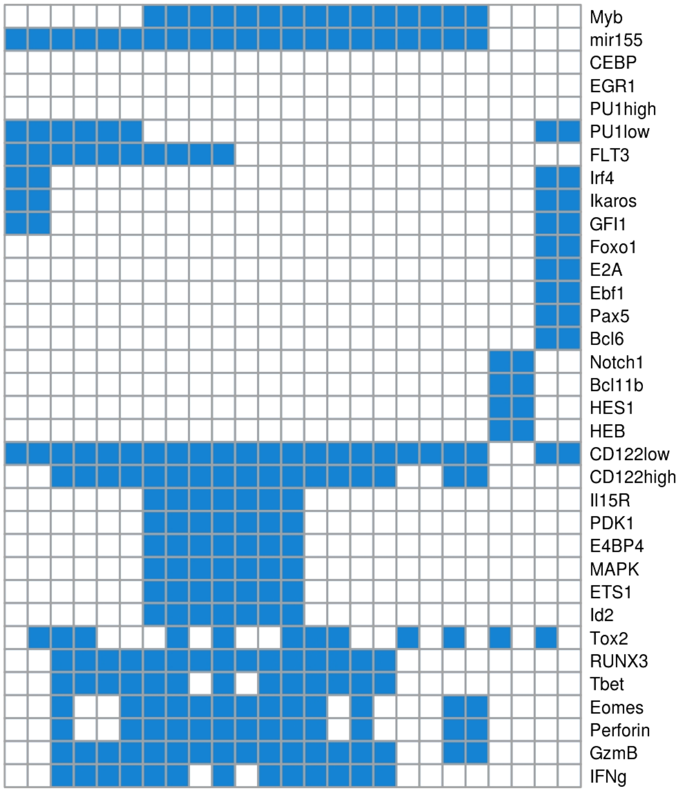


**Supplementary Figure 1. Steady states of a model variant that does not contain predicted regulatory interactions.** The fixed point attractors of this model variant, shown as columns, do not have correspondence with experimentally reported expression patterns. Dark and clear squares represent active and inactive nodes, respectively.

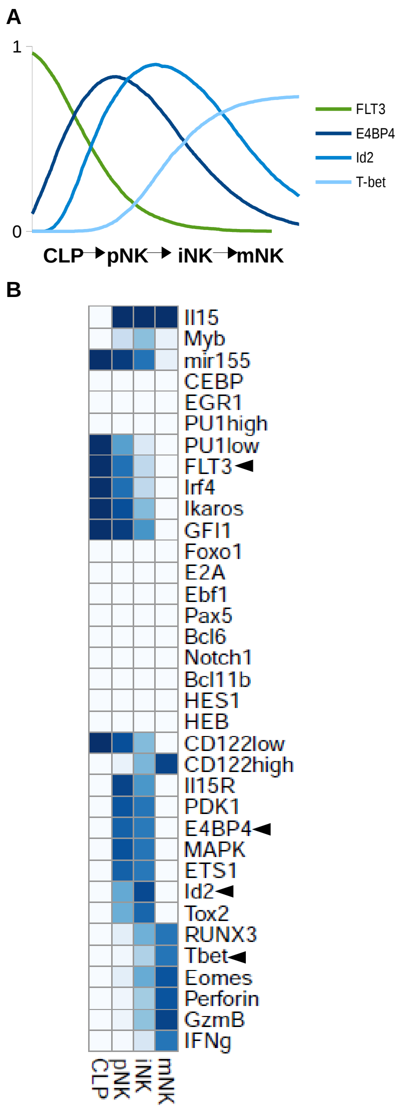


**Supplementary Figure 2. State transitions from CLP to mNK. A**. Average of 5000 state transitions of 4 genes that mark different cell states during NK differentiation: Flt3 that marks CLP, E4BP4 for pNK stage, Id2 for iNKs and T-bet for mNKs. **B**. Average of transitions in four different time-steps during simulation. The distinct states correlate with intermediate cells of the process (pNK and iNK) until evolve to a stationary state (mNK). Marked with arrow heads the genes plotted in A.

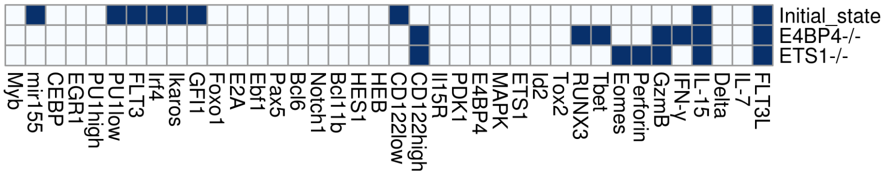


**Supplementary Figure 3. Simulation of E4BP4 and ETS1 knockouts.** Attractors of the model after simulation of E4BP4 or ETS1 knockouts (node value=0 during simulation). Simulation was performed starting from CLP attractor state in addition with IL-15 and FLT3L. White squares mean absence of genes, and blue genes present or active.

| **Gene** | **Interaction** | **Target** | **References** |
|---|---|---|---|
| Myb | Activation | miR-155 | Basova et al. [2013], Vargova et al. [2011] |
| miR-155 | Inhibition | Pu.1 | Vigorito et al. [2007], O'Connell et al. [2008], Basova et al. [2013], Ger |
| CEBPa | Activation | Pu.1 | Yeamans et al. [2007] |
| EGR1 | Activation | Pu.1 | Spooner et al. [2009] |
| EGR1 | Inhibition | Gfi1 | Spooner et al. [2009] |
| EGR1 | Activation | Id2 | Spooner et al. [2009] |
| Flt3L | Activation | Flt3 | Hannum et al. [1994] |
| Flt3 | Activation | miR-155 | Gerloff et al. [2015] |
| Flt3 | Inhibition | CEBPa | Zheng et al. [2004] |
| Flt3 | Activation | Pu.1 | Onai et al. [2006] |
| Flt3 | Activation | CD122 | Yu et al. [1998] |
| PU.1 | Activation | Pu.1 | Okuno et al. [2005] |
| PU.1 | Inhibition | Myb | Basova et al. [2013] |
| PU.1 | Activation | CEBPa | Kamath et al. [2008], Spooner et al. [2009] |
| PU.1 | Activation | EGR1 | Spooner et al. [2009] |
| PU.1 | Dual | Flt3 | Inomata et al. [2006], Carotta et al. [2010] |
| PU.1 | Dual | Irf4 | Kamath et al. [2008] |
| PU.1 | Inhibition | Ikaros | Franco et al. [2006] |
| PU.1 | Activation | Ebf1 | Roessler et al. [2007] |
| PU.1 | Inhibition | Pax5 | Maitra and Atchison [2000] |
| PU.1 | Inhibition | Bcl11b | Franco et al. [2006] |
| PU.1 | Inhibition | HES-1 | Franco et al. [2006] |
| PU.1 | Inhibition | ETS1 | Franco et al. [2006] |
| PU.1 | Activation | Notch1 | Franco et al. [2006] |
| Irf4 | Inhibition | Pu.1 | Wang et al. [2015] |
| Irf4 | Activation | Ikaros | Ma et al. [2008] |
| Irf4 | Inhibition | RUNX3 | Cao et al. [2010] |
| Irf4 | Inhibition | Eomes | Nayar et al. [2012] |
| Ikaros | Inhibition | CEBPa | Rao et al. [2013] |
| Ikaros | Activation | Flt3 | Yoshida et al. [2006] |
| Ikaros | Dual | Pu.1 | Zarnegar and Rothenberg [2012] |
| Ikaros | Activation | Gfi1 | Spooner et al. [2009] |
| Ikaros | Inhibition | T-bet | Thomas et al. [2010] |
| Gfi1 | Inhibition | EGR1 | Spooner et al. [2009] |
| Gfi1 | Inhibition | Pu.1 | Dahl et al. [2006] |
| Gfi1 | Inhibition | Id2 | Li et al. [2010a], Spooner et al. [2009] |
| Il-7 | Activation | Ebf1 | Tsapogas et al. [2011] |
| Foxo1 | Inhibition | T-bet | Deng et al. [2015] |
| E2A | Activation | Gfi1 | Xu and Kee [2007] |
| E2A | Activation | Foxo1 | Lin et al. [2010] |
| E2A | Activation | Ebf1 | Smith et al. [2002], Roessler et al. [2007] |
| Ebf1 | Inhibition | CEBPa | Pongubala et al. [2008] |
| Ebf1 | Activation | Foxo1 | Lin et al. [2010] |
| Ebf1 | Activation | E2A | Zhuang et al. [2004], Lin et al. [2010], Roessler et al. [2007] |
| Ebf1 | Activation | Ebf1 | Smith et al. [2002], Roessler et al. [2007] |
| Ebf1 | Activation | Pax5 | O'Riordan and Grosschedl [1999] |
| Ebf1 | Activation | Bcl6 | Lin et al. [2010] |
| Ebf1 | Inhibition | Id2 | Thal et al. [2009] |
| Pax5 | Inhibition | Flt3 | Holmes et al. [2006] |
| Pax5 | Inhibition | Pu.1 | Maitra and Atchison [2000] |
| Pax5 | Activation | Ebf1 | Fuxa et al. [2004], Roessler et al. [2007] |
| Pax5 | Activation | Bcl6 | Nera et al. [2006] |
| Pax5 | Inhibition | Notch1 | Souabni et al. [2002] |

| | | | |
|---|---|---|---|
| Bcl6 | Inhibition | RUNX3 | Hatzi et al. [2015] |
| Bcl6 | Inhibition | T-bet | Hatzi et al. [2015] |
| Delta | Activation | Notch1 | Lai [2004] |
| Notch1 | Inhibition | Ebf1 | Taghon et al. [2005] |
| Notch1 | Activation | Notch1 | Yashiro-Ohtani et al. [2009] |
| Notch1 | Activation | Bcl11b | Li et al. [2010b] |
| Notch1 | Activation | HEB | Braunstein and Anderson [2011] |
| Notch1 | Activation | Hes1 | Taghon et al. [2005], Kato et al. [2015] |
| Notch1 | Activation | CD122 | Carotta et al. [2006]* |
| Hes1 | Inhibition | Flt3 | Kato et al. [2015] |
| Hes1 | Inhibition | Myb | Guiu et al. [2013] |
| Bcl11b | Inhibition | Flt3 | Bartram et al. [2014] |
| Bcl11b | Inhibition | Pu.1 | Ikawa et al. [2010] |
| Bcl11b | Activation | Gfi1 | Califano et al. [2015] |
| Bcl11b | Activation | Notch1 | Li et al. [2010b] |
| Bcl11b | Inhibition | E4BP4 | Li et al. [2010b] |
| Bcl11b | Inhibition | Id2 | Li et al. [2010b], Kastner et al. [2010] |
| Bcl11b | Inhibition | RUNX3 | Kastner et al. [2010] |
| Bcl11b | Inhibition | CD122 | Li et al. [2010b] |
| HEB | Activation | Gfi1 | Braunstein and Anderson [2011] |
| HEB | Inhibition | E2A | Wang et al. [2010] |
| HEB | Inhibition | Pax5 | Wang et al. [2010] |
| HEB | Activation | Notch1 | Braunstein and Anderson [2011] |
| Il-15 | Activation | Il15R | Williams et al. [1998] |
| Il15R | Activation | PDK-1 | Gascoyne et al. [2009], Yang et al. [2015] |
| Il15R | Activation | MAPK | Ramirez et al. [2012], Grund et al. [2005] |
| PDK1 | Inhibition | Foxo1 | Deng et al. [2015] |
| PDK1 | Activation | E4BP4 | Gascoyne et al. [2009], Yang et al. [2015] |
| E4BP4 | Activation | Id2 | Gascoyne et al. [2009], Male et al. [2014] |
| E4BP4 | Activation | Eomes | Male et al. [2014] |
| MAPK | Inhibition | Foxo1 | Deng et al. [2015] |
| MAPK | Activation | ETS1 | Ramirez et al. [2012], Grund et al. [2005] |
| ETS1 | Activation | Id2 | Ramirez et al. [2012], Zook et al. [2016] |
| ETS1 | Activation | RUNX3 | Zamisch et al. [2009] |
| ETS1 | Activation | T-bet | Ramirez et al. [2012] |
| ETS1 | Activation | CD122 | Ramirez et al. [2012] |
| ETS1 | Activation | Tox2 | Vong et al. [2014]* |
| Id2 | Inhibition | Pu.1 | Spooner et al. [2009] |
| Id2 | Inhibition | E2A | Boos et al. [2007] |
| Id2 | Inhibition | Pax5 | Gonda et al. [2003], Roberts et al. [2001] |
| Id2 | Inhibition | HEB | Schotte et al. [2010] |
| RUNX3 | Activation | CD122 | Lai and Mager [2012] |
| RUNX3 | Inhibition | Notch1 | Gao et al. [2010] |
| RUNX3 | Activation | Eomes | Cruz-Guilloty et al. [2009] |
| RUNX3 | Activation | IFN-g | Djuretic et al. [2007] |
| Tox2 | Activation | T-bet | Vong et al. [2014] |
| T-bet | Inhibition | Irf4 | Gökmen et al. [2013] |
| T-bet | Inhibition | Bcl6 | Nakayamada et al. [2011] |
| T-bet | Activation | RUNX3 | Reis et al. [2014], Djuretic et al. [2007] |
| T-bet | Activation | T-bet | Kanhere et al. [2012] |
| T-bet | Activation | CD122 | Intlekofer et al. [2005] |
| T-bet | Activation | Granzyme B | Daussy et al. [2014] |
| T-bet | Activation | IFN-g | Djuretic et al. [2007] |
| Eomes | Activation | Eomes | Kartikasari et al. [2013], Kidder and Palmer [2010] |

| | | | |
|---|---|---|---|
| Eomes | Activation | CD122 | Intlekofer et al. [2005] |
| Eomes | Activation | Granzyme B | Knox et al. [2014], Daussy et al. [2014] |
| Eomes | Activation | Perforin | Knox et al. [2014] |
| CD122 | Inhibition | Flt3 | Lau-Kilby et al. [2011]* |
| CD122 | Activation | Il15R | Carson et al. [1994] |
| CD122 | Inhibition | Myb | Sullivan et al. [2015]* |
| Granzyme B | Inhibition | Notch1 | van Tetering et al. [2011] |

**Supplementary Table 1**. List of interactions (activations, inhibitions and dual) among the 36 molecules that form the NK regulatory network and the bibliography that supports them, mainly related to NK cells or hematopoietic process in mice and human. Asterisks indicate the references that support the predicted interactions of the model.

| Target | Regulatory Rule |
|---|---|
| Myb | ¬(PU1high∨PU1low∨HES1∨CD122high) |
| miR155 | Myb∨Flt3 |
| CEBPα | PU1high ∧ ¬Ikaros ∧ ¬Ebf1 ∧ ¬Flt3 |
| EGR1 | PU1high ∧ ¬GFI1 |
| PU1high | ¬(Bcl11b∨miR155∨Pax5∨GFI1∨Irf4∨Ikaros∨Id2)∧ (EGR1∨PU1low∨PU1high) ∧ CEBPα |
| PU1low | ¬(Bcl11b∨Id2)∧ (Flt3∨(PU1low∧Ikaros)) |
| Flt3 | ¬(HES1∨Bcl11b∨CD122high∨Pax5∨PU1high)∧((Ikaros∧PU1low)∨Flt3l) |
| Irf4 | ¬(Tbet∨PU1high)∧PU1low |
| Ikaros | Irf4 ∧ ¬PU1high |
| GFI1 | ¬EGR1∧(Ikaros∨(HEB∨Bcl11b)∧E2A) |
| Foxo1 | ¬(PDK1∨MAPK) ∧ (Ebf1∨E2A) |
| E2A | Ebf1∧¬HEB∧¬Id2 |
| Ebf1 | ¬Notch1 ∧ PU1low ∧ ((Ebf1∧E2A∧Pax5) ∨ Il7) |
| Pax5 | Ebf1 ∧ ¬(Id2∨HEB∨PU1high) |
| Bcl6 | (Pax5∨Ebf1) ∧ ¬Tbet |
| Notch1 | ((Delta ∧ PU1low) ∨ (Notch1 ∧ Bcl11b ∧ HEB))∧¬(Pax5∨RUNX3∨GzmB) |
| Bcl11b | Notch1∧¬PU1high |
| HES1 | Notch1∧¬PU1high |
| HEB | Notch1 ∧¬Id2 |
| CD122low | ¬Bcl11b ∧ (Flt3∨Notch1) |
| CD122high | ¬Bcl11b ∧ (Tbet ∨ Eomes ∨ (ETS1∧RUNX3)) |
| Il15R | Il15 ∧ CD122low |
| PDK1 | Il15R |
| E4BP4 | PDK1∧¬Bcl11b |
| MAPK | Il15R |
| ETS1 | MAPK∨(¬PU1low∧¬PU1high∧MAPK) |
| Id2 | ¬Bcl11b ∧ ((¬Ebf1 ∧ ¬GFI1 ∧ EGR1) ∨ E4BP4 ∨ ETS1) |
| Tox2 | ETS1 |
| RUNX3 | ¬(Irf4∨Bcl11b∨Bcl6)∧(ETS1∨Tbet) |
| Tbet | ¬Ikaros ∧ ¬Bcl6 ∧ ¬Foxo1 ∧ ((ETS1 ∧ Tox2) ∨ Tbet) |
| Eomes | ¬Irf4 ∧ (E4BP4 ∨ (E4BP4 ∧ RUNX3) ∨ (Eomes ∧ RUNX3) ∨ Eomes) |
| Perforin | Eomes |
| GzmB | Tbet∨Eomes |
| IFN-γ | Tbet∧RUNX3 |

**Supplementary Table 2.** The rules determining the state of activation of each node as a function of its regulatory inputs. They are expressed with the use of the logic operators ∧ (AND), ∨ (OR), and ¬ (NOT).

| | Genetic supression | | Over-expression | | |
|---|---|---|---|---|---|
| Gene | Model results (node=0) | Experimental results | Model results (node=1) | Experimental results | References |
| | Initial state 0 | | | | |
| Myb | - | Loss HSC | HSC Cebp+ | | Baker S., et al., 2014; Lieu Y. and Reddy P., 2009 |
| miR-155 | - | Cell Differentiation | HSC Cebp+ | CMP disorder | Havelange V. and Garzon R., 2010; O'Connell R. et al., 2008 |
| | Initial state HSC+Flt3L | | | | |
| Flt3 | HSC | Loss of CLP, ETP and proB | $Flt3^{+}PU.1^{+}RUNX3^{+}$ | Acute Myeloid Leukemia ($Flt3^{+}RUNX3^{+}$) | Gwin K. et al., 2013; Damdinsuren A. et al. 2015 |
| PU.1 | HSC | Loss of CLP | CMP | Loss of CLP and CMP differentiation | Iwasaki H. et al., 2005; Mclvor Z. et al., 2003 |
| Ikaros | CLP | Loss of CLP and NK | Loss of NK 1 and NK 2 | | Boggs et al., 1998 |
| Irf4 | Loss of CLP | Loss of CLP; CMP differentiation | Loss of NK 1, 2 and 3 | CLP differentiation | Wang S. et al., 2015 |
| GFI1 | - | Loss of CLP and proB | - | proB differentiation | Spooner C. et al., 2009 |
| | Initial state proB +IL-15 | | | | |
| E2A | reprogramming to NK | Loss of proB, NK differentiation | Loss of NK 1 and NK 2 | | Boos M. et al., 2007 |
| Ebf1 | reprogramming to NK | Loss of proB | Loss of CLP, NK 1 and NK 2; proB differentiation | proB differentiation | Medina K. et al., 2004 |
| Pax5 | reprogramming to NK | proB reprogramming to NK | Loss of ETP | proB differentiation | Souabni A. et al., 2002; Carotta S. et al. 2006 |
| Foxo1 | - | Loss of proB | Loss of NK 1 and NK 2 | proB differentiation | Gubbels M. et al., 2009 |
| | Initial state ETP +IL-15 | | | | |
| Notch1 | reprogramming to NK | | ETP differentiation only | T lymphocyte differentiation | Braunstein and Anderson 2011 |
| Bcl11b | reprogramming to NK | ETP reprogramming to NK | ETP and NK Differentiation | | Li P. et al., 2010 |
| HEB | reprogramming to NK | ETP reprogramming to NK | Loss of proB | T lymphocyte differentiation | Barndt R. et al., 2000; Braunstein and Anderson 2011 |
| | Initial state CLP +IL-15 | | | | |
| CD122 | CLP | Loss ok NK | Loss of HSC | | Sevilir N. et al., 1998 |
| IL-15R | CLP | Loss of pNK | NK differentiation only | | Sevilir N. et al., 1998 |
| E4BP4 | Loss of NK 2 and 3 | Loss of pNK | NK differentiation only | NK differentiation | Male V. et al., 2014; Yang M. et al., 2015 |
| ETS1 | Loss of NK 1 and 2 | NK decrease | NK differentiation only | | Barton K. et al., 1998 |
| Id2 | pNK | Loss of NK | Loss of CLP, proB and ETP | Loss of proB and ETP | Thai M. et al., 2009; Huajie L. et al., 2010; Boos M. et al., 2007 |
| Tox2 | Loss of NK 1 and 2 | Loss of NK | - | | Vong Q. et al., 2015 |
| RUNX3 | - | Loss of NK | Loss of ETP | | Levanon et al., 2014 |
| T-bet | Loss of NK 1 and 2 | Loss of NK | NK differentiation only | | Vong Q. et al., 2015 |
| Eomes | Loss of NK 2 and 3 | Loss of NK | Loss of ETP | NK differentiation | Male V. et al., 2014; Gordon S. et al., 2012 |

**Supplementary Table 3.** Simulation of genetic loss (node=0) and gain (node=1) of function mutants compared against experimental observations. Shaded rows indicate congruence between our model and the experimental results. For loss of function analysis a specific initial state, resembling experimental probes, was used. Outputs, one input/one output, constantly inactivated and multi-level nodes are not presented due to its redundancy over the results. Loss of function simulations were performed starting from specific initial states and extracellular signals in order to compare with experimental reports.

# References


P Basova, V Pospisil, F Savvulidi, P Burda, K Vargova, L Stanek, M Dluhosova, E Kuzmova, A Jonasova, U Steidl, P Laslo, and T Stopka. Aggressive acute myeloid leukemia in PU.1/p53 double-mutant mice. *Oncogene*, 33 (39):4735–4745, 2013. ISSN 1476-5594. doi: 10.1038/onc.2013.414. URL `http://dx.doi.org/10.1038/onc.2013.414`.

Karin Vargova, Nikola Curik, Pavel Burda, Petra Basova, Vojtech Kulvait, Vit Pospisil, Filipp Savvulidi, Juraj Kokavec, Emanuel Necas, Adela Berkova, Petra Obrtlikova, Josef Karban, Marek Mraz, Sarka Pospisilova, Jiri Mayer, Marek Trneny, Jiri Zavadil, and Tomas Stopka. Myb transcriptionally regulates the mir-155 host gene in chronic lymphocytic leukemia. *Blood*, 117(14):3816–3825, 2011. ISSN 0006-4971. doi: 10.1182/blood-2010-05-285064. URL `http://www.bloodjournal.org/content/117/14/3816`.

Elena Vigorito, Kerry L. Perks, Cei Abreu-Goodger, Sam Bunting, Zou Xiang, Susan Kohlhaas, Partha P. Das, Eric A. Miska, Antony Rodriguez, Allan Bradley, Kenneth G C Smith, Cristina Rada, Anton J. Enright, Kai Michael Toellner, Ian C M MacLennan, and Martin Turner. microRNA-155 Regulates the Generation of Immunoglobulin Class-Switched Plasma Cells. *Immunity*, 27(6):847–859, 2007. ISSN 10747613. doi: 10.1016/j.immuni.2007.10.009.

Ryan M. O'Connell, Dinesh S. Rao, Aadel A. Chaudhuri, Mark P. Boldin, Konstantin D. Taganov, John Nicoll, Ronald L. Paquette, and David Baltimore. Sustained expression of microRNA-155 in hematopoietic stem cells causes a myeloproliferative disorder. *The Journal of Experimental Medicine*, 205 (3):585–594, 2008. ISSN 0022-1007. doi: 10.1084/jem.20072108. URL `http://www.jem.org/lookup/doi/10.1084/jem.20072108`.

D Gerloff, R Grundler, A A Wurm, D Bräuer-Hartmann, C Katzerke, J-U Hartmann, V Madan, C Müller-Tidow, J Duyster, D G Tenen, D Niederwieser, and G Behre. NF-$\kappa$B/STAT5/miR-155 network targets PU.1 in FLT3-ITD-driven acute myeloid leukemia. *Leukemia*, 29(3):535–547, 2015. ISSN 0887-6924. doi: 10.1038/leu.2014.231. URL `http://www.nature.com/doifinder/10.1038/leu.2014.231`.

Christine Yeamans, Dehua Wang, Ido Paz-Priel, Bruce E. Torbett, Daniel G. Tenen, and Alan D. Friedman. C/EBP$\alpha$ binds and activates the PU.1 distal enhancer to induce monocyte lineage commitment. *Blood*, 110(9):3136–3142, 2007. ISSN 00064971. doi: 10.1182/blood-2007-03-080291.

Chauncey J. Spooner, Jason X. Cheng, Elisabet Pujadas, Peter Laslo, and Harinder Singh. A Recurrent Network Involving the Transcription Factors PU.1 and Gfi1 Orchestrates Innate and Adaptive Immune Cell Fates. *Immunity*, 31(4):576–586, 2009. ISSN 10747613. doi: 10.1016/j.immuni.2009.07.011. URL `http://dx.doi.org/10.1016/j.immuni.2009.07.011`.

C. Hannum, J. Culpepper, D. Campbell, T. McClanahan, S. Zurawski, J. F. Bazan, R. Kastelein, S. Hudak, J. Wagner, and J. Mattson. Ligand for FLT3/FLK2 receptor tyrosine kinase regulates growth of haematopoietic stem cells and is encoded by variant RNAs. *Nature*, 368(6472):643–648, Apr 1994.

Rui Zheng, Alan D. Friedman, Mark Levis, Li Li, Edward G. Weir, and Donald Small. Internal tandem duplication mutation of FLT3 blocks myeloid differentiation through suppression of C/EBP$\alpha$ expression. *Blood*, 103(5):1883–1890, 2004. ISSN 00064971. doi: 10.1182/blood-2003-06-1978.

Nobuyuki Onai, Aya Obata-Onai, Roxane Tussiwand, Antonio Lanzavecchia, and Markus G. Manz. Activation of the flt3 signal transduction cascade rescues and enhances type i interferon–producing and dendritic cell development. *Journal of Experimental Medicine*, 203(1):227–238, 2006. ISSN 0022-1007. doi: 10.1084/jem.20051645. URL http://jem.rupress.org/content/203/1/227.

H Yu, T a Fehniger, P Fuchshuber, K S Thiel, E Vivier, W E Carson, and M a Caligiuri. Flt3 ligand promotes the generation of a distinct CD34(+) human natural killer cell progenitor that responds to interleukin-15. *Blood*, 92 (10):3647–57, 1998. ISSN 0006-4971. URL http://www.ncbi.nlm.nih.gov/pubmed/9808558.

Yutaka Okuno, Gang Huang, Frank Rosenbauer, Erica K Evans, Hanna S Radomska, Hiromi Iwasaki, Koichi Akashi, Francoise Moreau-gachelin, Youlin Li, and Daniel G Tenen. Potential Autoregulation of Transcription Factor PU . 1 by an Upstream Regulatory Element. 25(7):2832–2845, 2005. doi: 10.1128/MCB.25.7.2832.

M B Kamath, I B Houston, A J Janovski, X Zhu, S Gowrisankar, A G Jegga, and R P DeKoter. Dose-dependent repression of T-cell and natural killer cell genes by PU.1 enforces myeloid and B-cell identity. *Leukemia*, 22(6): 1214–1225, 2008. ISSN 0887-6924. doi: 10.1038/leu.2008.67. URL http://www.nature.com/doifinder/10.1038/leu.2008.67.

Mitsue Inomata, Shinichiro Takahashi, Hideo Harigae, Junichi Kameoka, Mitsuo Kaku, Takeshi Sasaki, Sebastian Carotta, Aleksandar Dakic, Angela D'Amico, Swee Heng Milon Pang, Kylie T Greig, Stephen L Nutt, Li Wu, Chauncey J Spooner, Jason X Cheng, Elisabet Pujadas, Peter Laslo, Harinder Singh, Shinichiro Takahashi, Nobuyuki Onai, Aya Obata-Onai, Roxane Tussiwand, Antonio Lanzavecchia, Markus G Manz, and K Minton. Recipe for a B cell. *Immunity*, 4(1): 13, 2006. ISSN 10747613. doi: 10.1016/j.immuni.2010.05.005. URL http://www.nature.com/nri/journal/v4/n11/full/nri1506.htmlhttp://cdn.intechweb.org/pdfs/25047.pdfhttp://www.jhoonline.org/content/4/1/13http://dx.doi.org/10.1016/j.immuni.2009.07.011http://linkinghub.elsevier.com/retrieve/pii/S1074761310001718.

S. Carotta, A. Dakic, A. D'Amico, S. H. Pang, K. T. Greig, S. L. Nutt, and L. Wu. The transcription factor PU.1 controls dendritic cell development and

Flt3 cytokine receptor expression in a dose-dependent manner. *Immunity*, 32 (5):628–641, May 2010.

C. B. Franco, D. D. Scripture-Adams, I. Proekt, T. Taghon, A. H. Weiss, M. A. Yui, S. L. Adams, R. A. Diamond, and E. V. Rothenberg. Notch/Delta signaling constrains reengineering of pro-T cells by PU.1. *Proceedings of the National Academy of Sciences*, 103(32):11993–11998, 2006. ISSN 0027-8424. doi: 10.1073/pnas.0601188103. URL http://www.pnas.org/cgi/doi/10.1073/pnas.0601188103.

Stephanie Roessler, Ildiko Györy, Sascha Imhof, Mikhail Spivakov, Ruth R Williams, Meinrad Busslinger, Amanda G Fisher, and Rudolf Grosschedl. Distinct promoters mediate the regulation of Ebf1 gene expression by interleukin-7 and Pax5. *Molecular and cellular biology*, 27(2):579–594, 2007. ISSN 0270-7306. doi: 10.1128/MCB.01192-06. URL http://www.ncbi.nlm.nih.gov/pubmed/17101802.

S. Maitra and M. Atchison. BSAP Can Repress Enhancer Activity by Targeting PU.1 Function. *Molecular and Cellular Biology*, 20(6):1911–1922, 2000. ISSN 0270-7306. doi: 10.1128/MCB.20.6.1911-1922.2000. URL http://mcb.asm.org/cgi/doi/10.1128/MCB.20.6.1911-1922.2000.

Sifeng Wang, Qiuping He, Dongyuan Ma, Yuanyuan Xue, and Feng Liu. Irf4 Regulates the Choice between T Lymphoid-Primed Progenitor and Myeloid Lineage Fates during Embryogenesis. *Developmental Cell*, 34(6):621–631, 2015. ISSN 15345807. doi: 10.1016/j.devcel.2015.07.011. URL http://linkinghub.elsevier.com/retrieve/pii/S1534580715004621.

Shibin Ma, Simanta Pathak, Long Trinh, and Runqing Lu. Interferon regulatory factors 4 and 8 induce the expression of Ikaros and Aiolos to down-regulate pre-B-cell receptor and promote cell-cycle withdrawal in pre-B-cell development. *Blood*, 111(3):1396–1403, 2008. ISSN 00064971. doi: 10.1182/blood-2007-08-110106.

Yonghao Cao, Hai Li, Yang Sun, Xufeng Chen, Haifeng Liu, Xiang Gao, and Xiaolong Liu. Interferon regulatory factor 4 regulates thymocyte differentiation by repressing Runx3 expression. *European Journal of Immunology*, 40(11): 3198–3209, 2010. ISSN 00142980. doi: 10.1002/eji.201040570.

R. Nayar, M. Enos, A. Prince, H. Shin, S. Hemmers, J. K. Jiang, U. Klein, C. J. Thomas, and L. J. Berg. TCR signaling via Tec kinase ITK and interferon regulatory factor 4 (IRF4) regulates CD8+ T-cell differentiation. *Proc. Natl. Acad. Sci. U.S.A.*, 109(41):E2794–2802, Oct 2012.

K. N. Rao, C. Smuda, G. D. Gregory, B. Min, and M. A. Brown. Ikaros limits basophil development by suppressing C/EBP-expression. *Blood*, 122(15):2572–2581, 2013. ISSN 0006-4971. doi: 10.1182/blood-2013-04-494625. URL http://www.bloodjournal.org/cgi/doi/10.1182/blood-2013-04-494625.

Toshimi Yoshida, Samuel Yao-Ming Ng, Juan Carlos Zuniga-Pflucker, and Katia Georgopoulos. Early hematopoietic lineage restrictions directed by Ikaros. *Nature Immunology*, 7(4):382–391, 2006. ISSN 1529-2908. doi: 10.1038/ni1314. URL `http://www.nature.com/doifinder/10.1038/ni1314`.

M a Zarnegar and E V Rothenberg. Ikaros represses and activates PU.1 cell-type-specifically through the multifunctional Sfpi1 URE and a myeloid specific enhancer. *Oncogene*, 31(43):4647–4654, 2012. ISSN 0950-9232. doi: 10.1038/onc.2011.597. URL `http://dx.doi.org/10.1038/onc.2011.597`.

Rajan M. Thomas, Chunxia Chen, Neelanjana Chunder, Lingzhi Ma, Justin Taylor, Edward J. Pearce, and Andrew D. Wells. Ikaros silences T-bet expression and interferon-$\gamma$ production during T helper 2 differentiation. *Journal of Biological Chemistry*, 285(4):2545–2553, 2010. ISSN 00219258. doi: 10.1074/jbc.M109.038794.

R. Dahl, S. R. Iyer, K. S. Owens, D. D. Cuylear, and M. C. Simon. The Transcriptional Repressor GFI-1 Antagonizes PU.1 Activity through Protein-Protein Interaction. *Journal of Biological Chemistry*, 282(9):6473–6483, 2006. ISSN 0021-9258. doi: 10.1074/jbc.M607613200. URL `http://www.jbc.org/cgi/doi/10.1074/jbc.M607613200`.

Huajie Li, Ming Ji, Kimberly D Klarmann, and Jonathan R Keller. Repression of Id2 expression by Gfi-1 is required for B-cell and myeloid development. *Blood*, 116(7):1060–1069, 2010a. ISSN 1528-0020. doi: 10.1182/blood-2009-11-255075.

Panagiotis Tsapogas, Sasan Zandi, Josefine Åhsberg, Jenny Zetterblad, Eva Welinder, Jan Ingvar Jönsson, Robert Månsson, Hong Qian, and Mikael Sigvardsson. Il-7 mediates ebf-1–dependent lineage restriction in early lymphoid progenitors. *Blood*, 118(5):1283–1290, 2011. ISSN 0006-4971. doi: 10.1182/blood-2011-01-332189. URL `http://www.bloodjournal.org/content/118/5/1283`.

Yafei Youcai Deng, Yann Kerdiles, Jianhong Chu, Shunzong Yuan, Youwei Wang, Xilin Chen, Hsiaoyin Mao, Lingling Zhang, Jianying Zhang, Tiffany Hughes, Yafei Youcai Deng, Qi Zhang, Fangjie Wang, Xianghong Zou, Chang-Gong Liu, Aharon G Freud, Xiaohui Li, Michael A Caligiuri, Eric Vivier, and Jianhua Yu. Transcription factor Foxo1 is a negative regulator of natural killer cell maturation and function. *Immunity*, 42(3):457–470, 2015. ISSN 1097-4180 (Electronic). doi: 10.1016/j.immuni.2015.02.006. URL `http://dx.doi.org/10.1016/j.immuni.2015.02.006`.

Wei Xu and Barbara L. Kee. Growth factor independent 1B (Gfi1b) is an E2A target gene that modulates Gata3 in T-cell lymphomas. *Blood*, 109(10):4406–4414, 2007. ISSN 00064971. doi: 10.1182/blood-2006-08-043331.

Yin C Lin, Suchit Jhunjhunwala, Christopher Benner, Sven Heinz, Eva Welinder, Robert Mansson, Mikael Sigvardsson, James Hagman, Celso A Espinoza, Janusz Dutkowski, Trey Ideker, Christopher K Glass, and Cornelis Murre.

A global network of transcription factors, involving E2A, EBF1 and Foxo1, that orchestrates B cell fate. *Nature Immunology*, 11(7):635–643, 2010. ISSN 1529-2908. doi: 10.1038/ni.1891. URL `http://www.nature.com/doifinder/10.1038/ni.1891`.

E M Smith, R Gisler, and M Sigvardsson. Cloning and characterization of a promoter flanking the early B cell factor (EBF) gene indicates roles for E-proteins and autoregulation in the control of EBF expression. *J Immunol*, 169(1):261–270, 2002. ISSN 0022-1767. doi: 10.4049/jimmunol.169.1.261. URL `http://www.ncbi.nlm.nih.gov/pubmed/12077253`.

Jagan M R Pongubala, Daniel L Northrup, David W Lancki, Kay L Medina, Thomas Treiber, Eric Bertolino, Matthew Thomas, Rudolf Grosschedl, David Allman, and Harinder Singh. Transcription factor EBF restricts alternative lineage options and promotes B cell fate commitment independently of Pax5. *Nature Immunology*, 9(2):203–215, 2008. ISSN 1529-2908. doi: 10.1038/ni1555. URL `http://www.nature.com/doifinder/10.1038/ni1555`.

Yuan Zhuang, Annette Jackson, Lihua Pan, Kang Shen, and Meifang Dai. Regulation of E2A gene expression in B-lymphocyte development. *Molecular Immunology*, 40(16):1165–1177, 2004. ISSN 01615890. doi: 10.1016/j.molimm.2003.11.031.

Mary O'Riordan and Rudolf Grosschedl. Coordinate regulation of B cell differentiation by the transcription factors EBF and E2A. *Immunity*, 11(1):21–31, 1999. ISSN 10747613. doi: 10.1016/S1074-7613(00)80078-3.

Melissa A Thal, Thiago L Carvalho, Ti He, Hyung-Gyoon Kim, Hua Gao, James Hagman, and Christopher A Klug. Ebf1-mediated down-regulation of Id2 and Id3 is essential for specification of the B cell lineage. *Proceedings of the National Academy of Sciences of the United States of America*, 106 (2):552–7, 2009. ISSN 1091-6490. doi: 10.1073/pnas.0802550106. URL `http://www.ncbi.nlm.nih.gov/pubmed/19122139`.

M. L. Holmes, S. Carotta, L. M. Corcoran, and S. L. Nutt. Repression of Flt3 by Pax5 is crucial for B-cell lineage commitment. *Genes Dev.*, 20(8):933–938, Apr 2006.

Martin Fuxa, Jane Skok, Abdallah Souabni, Giorgia Salvagiotto, Esther Roldan, and Meinrad Busslinger. Pax5 induces V-to-DJ rearrangements and locus contraction of the immunoglobulin heavy-chain gene. *Genes & Development*, (D):411–422, 2004. doi: 10.1101/gad.291504.action.

Kalle Pekka Nera, Pekka Kohonen, Elli Narvi, Anne Peippo, Laura Mustonen, Perttu Terho, Kimmo Koskela, Jean Marie Buerstedde, and Olli Lassila. Loss of Pax5 promotes plasma cell differentiation. *Immunity*, 24(3):283–293, 2006. ISSN 10747613. doi: 10.1016/j.immuni.2006.02.003.

Abdallah Souabni, Cesar Cobaleda, Michael Schebesta, and Meinrad Busslinger. Pax5 promotes B lymphopoiesis and blocks T cell development by repressing Notch1. *Immunity*, 17:781–793, 2002. ISSN 10747613. doi: 10.1016/S1074-7613(02)00472-7.

Katerina Hatzi, J. Philip Nance, Mark A. Kroenke, Marcella Bothwell, Elias K. Haddad, Ari Melnick, and Shane Crotty. BCL6 orchestrates Tfh cell differentiation via multiple distinct mechanisms. *The Journal of Experimental Medicine*, 212(4):539–553, 2015. ISSN 0022-1007. doi: 10.1084/jem.20141380. URL http://www.jem.org/lookup/doi/10.1084/jem.20141380.

Eric C. Lai. Notch signaling: control of cell communication and cell fate. *Development*, 131(5):965–973, 2004. ISSN 0950-1991. doi: 10.1242/dev.01074. URL http://dev.biologists.org/content/131/5/965.

Tom N. Taghon, Elizabeth Sharon David, Juan Carlos Zúñiga-Pflücker, and Ellen V. Rothenberg. Delayed, asynchronous, and reversible T-lineage specification induced by Notch/Delta signaling. *Genes and Development*, 19(8): 965–978, 2005. ISSN 08909369. doi: 10.1101/gad.1298305.

Yumi Yashiro-Ohtani, Yiping He, Takuya Ohtani, Mary E. Jones, Olga Shestova, Lanwei Xu, Terry C. Fang, Mark Y. Chiang, Andrew M. Intlekofer, Stephen C. Blacklow, Yuan Zhuang, and Warren S. Pear. Pre-TCR signaling inactivates Notch1 transcription by antagonizing E2A. *Genes and Development*, 23(14): 1665–1676, 2009. ISSN 08909369. doi: 10.1101/gad.1793709.

P. Li, S. Burke, J. Wang, X. Chen, M. Ortiz, S.-C. Lee, D. Lu, L. Campos, D. Goulding, B. L. Ng, G. Dougan, B. Huntly, B. Gottgens, N. A. Jenkins, N. G. Copeland, F. Colucci, and P. Liu. Reprogramming of T Cells to Natural Killer-Like Cells upon Bcl11b Deletion. *Science*, 329(5987): 85–89, 2010b. ISSN 0036-8075. doi: 10.1126/science.1188063. URL http://www.sciencemag.org/content/329/5987/85.abstract{%}5Cnhttp://www.sciencemag.org/content/329/5987/85.figures-only.

Marsela Braunstein and Michele K Anderson. HEB-deficient T-cell precursors lose T-cell potential and adopt an alternative pathway of differentiation. *Molecular and cellular biology*, 31(5):971–82, 2011. ISSN 1098-5549. doi: 10.1128/MCB.01034-10. URL http://www.pubmedcentral.nih.gov/articlerender.fcgi?artid=3067813{&}tool=pmcentrez{&}rendertype=abstract.

T Kato, M Sakata-Yanagimoto, H Nishikii, M Ueno, Y Miyake, Y Yokoyama, Y Asabe, Y Kamada, H Muto, N Obara, K Suzukawa, Y Hasegawa, I Kitabayashi, K Uchida, A Hirao, H Yagita, R Kageyama, and S Chiba. Hes1 suppresses acute myeloid leukemia development through FLT3 repression. *Leukemia*, 29(3):576–585, 2015. ISSN 0887-6924. doi: 10.1038/leu.2014.281. URL http://www.nature.com/doifinder/10.1038/leu.2014.281.

Sebastian Carotta, Jason Brady, Li Wu, and Stephen L. Nutt. Transient notch signaling induces nk cell potential in pax5-deficient pro-b cells. *European*

*Journal of Immunology*, 36(12):3294–3304, 2006. ISSN 1521-4141. doi: 10.1002/eji.200636325. URL http://dx.doi.org/10.1002/eji.200636325.

J. Guiu, R. Shimizu, T. D'Altri, S. T. Fraser, J. Hatakeyama, E. H. Bresnick, R. Kageyama, E. Dzierzak, M. Yamamoto, L. Espinosa, and A. Bigas. Hes repressors are essential regulators of hematopoietic stem cell development downstream of Notch signaling. *J. Exp. Med.*, 210(1):71–84, Jan 2013.

Isabelle Bartram, Nicola Gökbuget, Cornelia Schlee, Sandra Heesch, Lars Fransecky, Stefan Schwartz, Reingard Stuhlmann, Kerstin Schäfer-Eckhart, Michael Starck, Albrecht Reichle, Dieter Hoelzer, Claudia D Baldus, and Martin Neumann. Low expression of T-cell transcription factor BCL11b predicts inferior survival in adult standard risk T-cell acute lymphoblastic leukemia patients. *Journal of Hematology & Oncology*, 7(1):51, 2014. ISSN 1756-8722. doi: 10.1186/s13045-014-0051-y. URL http://jhoonline.biomedcentral.com/articles/10.1186/s13045-014-0051-y.

T. Ikawa, S. Hirose, K. Masuda, K. Kakugawa, R. Satoh, A. Shibano-Satoh, R. Kominami, Y. Katsura, and H. Kawamoto. An Essential Developmental Checkpoint for Production of the T Cell Lineage. *Science*, 329(5987):93–96, 2010. ISSN 0036-8075. doi: 10.1126/science.1188995. URL http://www.sciencemag.org/cgi/doi/10.1126/science.1188995.

Danielle Califano, Jonathan J. Cho, Mohammad N. Uddin, Kyle J. Lorentsen, Qi Yang, Avinash Bhandoola, Hongmin Li, and Dorina Avram. Transcription Factor Bcl11b Controls Identity and Function of Mature Type 2 Innate Lymphoid Cells. *Immunity*, 43(2):354–368, 2015. ISSN 10974180. doi: 10.1016/j.immuni.2015.07.005. URL http://dx.doi.org/10.1016/j.immuni.2015.07.005.

Philippe Kastner, Susan Chan, Walter K Vogel, Ling Juan Zhang, Acharawan Topark-Ngarm, Olga Golonzhka, Bernard Jost, Stéphanie Le Gras, Michael K Gross, and Mark Leid. Bcl11b represses a mature T-cell gene expression program in immature CD4+CD8+ thymocytes. *European Journal of Immunology*, 40(8):2143–2154, 2010. ISSN 00142980. doi: 10.1002/eji.200940258.

D Wang, C L Claus, P Rajkumar, M Braunstein, A J Moore, M Sigvardsson, and M K Anderson. Context-dependent regulation of hematopoietic lineage choice by HEBAlt. *J Immunol*, 185(7):4109–4117, 2010. ISSN 1550-6606. doi: 10.4049/jimmunol.0901783.

N S Williams, J Klem, I J Puzanov, P V Sivakumar, J D Schatzle, M Bennett, and V Kumar. Natural killer cell differentiation: insights from knockout and transgenic mouse models and in vitro systems. *Immunological reviews*, 165: 47–61, 1998. ISSN 0105-2896. doi: 10.1111/j.1600-065X.1998.tb01229.x.

Duncan M Gascoyne, Elaine Long, Henrique Veiga-Fernandes, Jasper de Boer, Owen Williams, Benedict Seddon, Mark Coles, Dimitris Kioussis, and Hugh J M Brady. The basic leucine zipper transcription factor E4BP4 is essential

for natural killer cell development. *Nature immunology*, 10(10):1118–1124, 2009. ISSN 1529-2908. doi: 10.1038/ni.1787.

Meixiang Yang, Dan Li, Zai Chang, Zhongzhou Yang, Zhigang Tian, and Zhongjun Dong. PDK1 orchestrates early NK cell development through induction of E4BP4 expression and maintenance of IL-15 responsiveness. *The Journal of experimental medicine*, 212(2):253–265, 2015. ISSN 1540-9538. doi: 10.1084/jem.20141703. URL http://www.ncbi.nlm.nih.gov/pubmed/25624444.

Kevin Ramirez, Katherine J. Chandler, Christina Spaulding, Sasan Zandi, Mikael Sigvardsson, Barbara J. Graves, and Barbara L. Kee. Gene Deregulation and Chronic Activation in Natural Killer Cells Deficient in the Transcription Factor ETS1. *Immunity*, 36(6):921–932, 2012. ISSN 10747613. doi: 10.1016/j.immuni.2012.04.006. URL http://dx.doi.org/10.1016/j.immuni.2012.04.006.

Eric M. Grund, Demetri D. Spyropoulos, Dennis K. Watson, and Robin C. Muise-Helmericks. Interleukins 2 and 15 regulate Ets1 expression via ERK1/2 and MNK1 in human natural killer cells. *Journal of Biological Chemistry*, 280 (6):4772–4778, 2005. ISSN 00219258. doi: 10.1074/jbc.M408356200.

Victoria Male, Ilaria Nisoli, Tomasz Kostrzewski, David S J Allan, James R Carlyle, Graham M Lord, Andreas Wack, and Hugh J M Brady. The transcription factor E4bp4/Nfil3 controls commitment to the NK lineage and directly regulates Eomes and Id2 expression. *The Journal of experimental medicine*, 211(4):635–42, 2014. ISSN 1540-9538. doi: 10.1084/jem.20132398. URL http://www.pubmedcentral.nih.gov/articlerender.fcgi?artid=3978281{&}tool=pmcentrez{&}rendertype=abstract.

Erin C. Zook, Kevin Ramirez, Xiaohuan Guo, Grant van der Voort, Mikael Sigvardsson, Eric C. Svensson, Yang-Xin Fu, and Barbara L. Kee. The ETS1 transcription factor is required for the development and cytokine-induced expansion of ILC2. *The Journal of Experimental Medicine*, 213(5):687–696, 2016. ISSN 0022-1007. doi: 10.1084/jem.20150851. URL http://www.jem.org/lookup/doi/10.1084/jem.20150851.

Monica Zamisch, Linhua Tian, Roland Grenningloh, Yumei Xiong, Kathryn F. Wildt, Marc Ehlers, I-Cheng Ho, and Rémy Bosselut. The transcription factor Ets1 is important for CD4 repression and Runx3 up-regulation during CD8 T cell differentiation in the thymus. *The Journal of Experimental Medicine*, 206(12):2685–2699, 2009. ISSN 0022-1007. doi: 10.1084/jem.20092024. URL http://www.jem.org/lookup/doi/10.1084/jem.20092024.

Queenie P Vong, Wing Wai-hang Leung, Jim Houston, Ying Li, Barbara Rooney, Martha Holladay, Robert a J Oostendorp, and Wing Wai-hang Leung. TOX2 regulates human natural killer cell development by controlling T-BET expression. *Blood*, 124(26):3905–3914, 2014. ISSN 0006-4971, 1528-0020. doi: 10.1182/blood-2014-06-582965.The.

M D Boos, Y Yokota, G Eberl, and B L Kee. Mature natural killer cell and lymphoid tissue-inducing cell development requires Id2-mediated suppression of E protein activity. *J Exp Med*, 204 (5):1119–1130, 2007. ISSN 0022-1007. doi: 10.1084/jem.20061959. URL http://www.ncbi.nlm.nih.gov/pubmed/17452521{%}5Cnhttp://www.ncbi.nlm.nih.gov/pmc/articles/PMC2118569/pdf/jem2041119.pdf.

Hiroyuki Gonda, Manabu Sugai, Yukiko Nambu, Tomoya Katakai, Yasutoshi Agata, Kazuhiro J Mori, Yoshifumi Yokota, and Akira Shimizu. The balance between Pax5 and Id2 activities is the key to AID gene expression. *The Journal of experimental medicine*, 198(9):1427–1437, 2003. ISSN 0022-1007. doi: 10.1084/jem.20030802.

E Claire Roberts, Richard W Deed, Toshiaki Inoue, D John, Andrew D Sharrocks, and John D Norton. Id Helix-Loop-Helix Proteins Antagonize Pax Transcription Factor Activity by Inhibiting DNA Binding Id Helix-Loop-Helix Proteins Antagonize Pax Transcription Factor Activity by Inhibiting DNA Binding. 21(2):524–533, 2001. doi: 10.1128/MCB.21.2.524.

Remko Schotte, Wendy Dontje, Maho Nagasawa, Yuko Yasuda, Arjen Q Bakker, Hergen Spits, and Bianca Blom. Synergy between IL-15 and Id2 promotes the expansion of human NK progenitor cells, which can be counteracted by the E protein HEB required to drive T cell development. *Journal of immunology (Baltimore, Md. : 1950)*, 184(12):6670–9, 2010. ISSN 1550-6606. doi: 10.4049/jimmunol.0901508. URL http://www.jimmunol.org/content/184/12/6670.full.

C. B. Lai and D. L. Mager. Role of runt-related transcription factor 3 (RUNX3) in transcription regulation of natural cytotoxicity receptor 1 (NCR1/NKp46), an activating natural killer (NK) cell receptor. *J. Biol. Chem.*, 287(10):7324–7334, Mar 2012.

Juan Gao, Yu Chen, Kai Chun Wu, Jie Liu, Yan Qiu Zhao, Yang Lin Pan, Rui Du, Guo Rong Zheng, Yi Min Xiong, Hua Lin Xu, and Dai Ming Fan. RUNX3 directly interacts with intracellular domain of Notch1 and suppresses Notch signaling in hepatocellular carcinoma cells. *Experimental Cell Research*, 316 (2):149–157, 2010. ISSN 00144827. doi: 10.1016/j.yexcr.2009.09.025. URL http://dx.doi.org/10.1016/j.yexcr.2009.09.025.

Fernando Cruz-Guilloty, Matthew E. Pipkin, Ivana M. Djuretic, Ditsa Levanon, Joseph Lotem, Mathias G. Lichtenheld, Yoram Groner, and Anjana Rao. Runx3 and T-box proteins cooperate to establish the transcriptional program of effector CTLs. *The Journal of Experimental Medicine*, 206(1):51–59, 2009. ISSN 0022-1007. doi: 10.1084/jem.20081242. URL http://www.jem.org/lookup/doi/10.1084/jem.20081242.

Ivana M Djuretic, Ditsa Levanon, Varda Negreanu, Yoram Groner, Anjana Rao, and K Mark Ansel. Transcription factors T-bet and Runx3 cooperate to

activate Ifng and silence Il4 in T helper type 1 cells. *Nature Immunology*, 8(2):145–153, 2007. ISSN 1529-2908. doi: 10.1038/ni1424. URL http://www.nature.com/doifinder/10.1038/ni1424.

M. Refik Gökmen, Rong Dong, Aditi Kanhere, Nick Powell, Esperanza Perucha, Ian Jackson, Jane K. Howard, Maria Hernandez-Fuentes, Richard G. Jenner, and Graham M. Lord. Genome-wide regulatory analysis reveals that t-bet controls th17 lineage differentiation through direct suppression of irf4. *The Journal of Immunology*, pages 5925–5932, 2013. ISSN 0022-1767. doi: 10.4049/jimmunol.1202254. URL http://www.jimmunol.org/content/early/2013/11/17/jimmunol.1202254.

Shingo Nakayamada, Yuka Kanno, Hayato Takahashi, Dragana Jankovic, Kristina T. Lu, Thomas A. Johnson, Hong wei Sun, Golnaz Vahedi, Ofir Hakim, Robin Handon, Pamela L. Schwartzberg, Gordon L. Hager, and John J. O'Shea. Early Th1 Cell Differentiation Is Marked by a Tfh Cell-like Transition. *Immunity*, 35(6):919–931, 2011. ISSN 10747613. doi: 10.1016/j.immuni.2011.11.012. URL http://dx.doi.org/10.1016/j.immuni.2011.11.012.

Bernardo S. Reis, David P. Hoytema van Konijnenburg, Sergei I. Grivennikov, and Daniel Mucida. Transcription factor T-bet regulates intraepithelial lymphocyte functional maturation. *Immunity*, 41(2):244–256, 2014. ISSN 10974180. doi: 10.1016/j.immuni.2014.06.017. URL http://dx.doi.org/10.1016/j.immuni.2014.06.017.

Aditi Kanhere, Arnulf Hertweck, Urvashi Bhatia, M. Refik Gökmen, Esperanza Perucha, Ian Jackson, Graham M. Lord, and Richard G. Jenner. T-bet and GATA3 orchestrate Th1 and Th2 differentiation through lineage-specific targeting of distal regulatory elements. *Nature Communications*, 3:1268, 2012. ISSN 2041-1723. doi: 10.1038/ncomms2260. URL http://www.nature.com/doifinder/10.1038/ncomms2260.

Andrew M Intlekofer, Naofumi Takemoto, E John Wherry, Sarah a Longworth, John T Northrup, Vikram R Palanivel, Alan C Mullen, Christopher R Gasink, Susan M Kaech, Joseph D Miller, Laurent Gapin, Kenneth Ryan, Andreas P Russ, Tullia Lindsten, Jordan S Orange, Ananda W Goldrath, Rafi Ahmed, and Steven L Reiner. Effector and memory CD8+ T cell fate coupled by T-bet and eomesodermin. *Nature immunology*, 6(12):1236–1244, 2005. ISSN 1529-2908. doi: 10.1038/ni1268.

Cécile Daussy, Fabrice Faure, Katia Mayol, Sébastien Viel, Georg Gasteiger, Emily Charrier, Jacques Bienvenu, Thomas Henry, Emilie Debien, Uzma A Hasan, Jacqueline Marvel, Keigyou Yoh, Satoru Takahashi, Immo Prinz, Simon de Bernard, Laurent Buffat, and Thierry Walzer. T-bet and Eomes instruct the development of two distinct natural killer cell lineages in the liver and in the bone marrow. *The Journal of experimental medicine*, 211(3):563–77, 2014. ISSN 1540-9538. doi: 10.1084/

jem.20131560. URL http://www.pubmedcentral.nih.gov/articlerender.fcgi?artid=3949572{&}tool=pmcentrez{&}rendertype=abstract.

Apriliana E R Kartikasari, Josie X Zhou, Murtaza S Kanji, David N Chan, Arjun Sinha, Anne Grapin-Botton, Mark A Magnuson, William E Lowry, and Anil Bhushan. The histone demethylase Jmjd3 sequentially associates with the transcription factors Tbx3 and Eomes to drive endoderm differentiation. *The EMBO Journal*, 32(10):1393–1408, 2013. ISSN 0261-4189. doi: 10.1038/emboj.2013.78. URL http://emboj.embopress.org/cgi/doi/10.1038/emboj.2013.78.

B. L. Kidder and S. Palmer. Examination of transcriptional networks reveals an important role for TCFAP2C, SMARCA4, and EOMES in trophoblast stem cell maintenance. *Genome Res.*, 20(4):458–472, Apr 2010.

J. J. Knox, G. L. Cosma, M. R. Betts, and L. M. McLane. Characterization of T-bet and eomes in peripheral human immune cells. *Front Immunol*, 5:217, 2014.

Annie W A.W. Lau-Kilby, Cosima C.C. Kretz, Susanne Pechhold, Jeffrey D J.D. Price, Stephanie Dorta, Haydee Ramos, G. Trinchieri, and K.V. Tarbell. Interleukin-2 inhibits FMS-like tyrosine kinase 3 receptor ligand (flt3L)-dependent development and function of conventional and plasmacytoid dendritic cells. *Proceedings of the National Academy of Sciences*, 108(6):2408, 2011. doi: 10.1073/pnas.1009738108/-/DCSupplemental.www.pnas.org/cgi/doi/10.1073/pnas.1009738108. URL http://www.pnas.org/content/108/6/2408.short.

W E Carson, J G Giri, M J Lindemann, M L Linett, M Ahdieh, R Paxton, D Anderson, J Eisenmann, K Grabstein, and M a Caligiuri. Interleukin (IL) 15 is a novel cytokine that activates human natural killer cells via components of the IL-2 receptor. *The Journal of experimental medicine*, 180(4):1395–1403, 1994. ISSN 0022-1007. doi: 10.1084/jem.180.4.1395.

Ryan P. Sullivan, Jeffrey W. Leong, Stephanie E. Schneider, Aaron R. Ireland, Melissa M. Berrien-Elliott, Anvita Singh, Timothy Schappe, Brea A. Jewell, Veronika Sexl, and Todd A. Fehniger. Microrna-15/16 antagonizes myb to control nk cell maturation. *The Journal of Immunology*, 195(6):2806–2817, 2015. ISSN 0022-1767. doi: 10.4049/jimmunol.1500949. URL http://www.jimmunol.org/content/195/6/2806.

Geert van Tetering, Niels Bovenschen, Jan Meeldijk, Paul J. van Diest, and Marc Vooijs. Cleavage of Notch1 by granzyme B disables its transcriptional activity. *Biochemical Journal*, 437(2):313–322, 2011. ISSN 0264-6021. doi: 10.1042/BJ20110226. URL http://biochemj.org/lookup/doi/10.1042/BJ20110226.
